# Orientationally-averaged diffusion-attenuated magnetic resonance signal for locally-anisotropic diffusion

**DOI:** 10.1038/s41598-019-41317-8

**Published:** 2019-03-20

**Authors:** Magnus Herberthson, Cem Yolcu, Hans Knutsson, Carl-Fredrik Westin, Evren Özarslan

**Affiliations:** 1 Department of Mathematics, Linköping University, Linköping, Sweden; 2 Department of Biomedical Engineering, Linköping University, Linköping, Sweden; 3 Center for Medical Image Science and Visualization, Linköping University, Linköping, Sweden; 4 Laboratory for Mathematics in Imaging, Department of Radiology, Brigham and Women’s Hospital, Harvard Medical School, Boston, MA USA

## Abstract

Diffusion-attenuated MR signal for heterogeneous media has been represented as a sum of signals from anisotropic Gaussian sub-domains to the extent that this approximation is permissible. Any effect of macroscopic (global or ensemble) anisotropy in the signal can be removed by averaging the signal values obtained by differently oriented experimental schemes. The resulting average signal is identical to what one would get if the micro-domains are isotropically (e.g., randomly) distributed with respect to orientation, which is the case for “powdered” specimens. We provide exact expressions for the orientationally-averaged signal obtained via general gradient waveforms when the microdomains are characterized by a general diffusion tensor possibly featuring three distinct eigenvalues. This extends earlier results which covered only axisymmetric diffusion as well as measurement tensors. Our results are expected to be useful in not only multidimensional diffusion MR but also solid-state NMR spectroscopy due to the mathematical similarities in the two fields.

## Introduction

In MR examinations of porous media as well as biological tissues, one is often confronted with a medium comprising an isotropic ensemble of individually anisotropic domains. The effect of diffusion within such media on the MR signal has thus been considered since the 70s^1–3^. The problem we tackle here is for the situation where diffusion within each microdomain can be taken to be free, thus can be characterized by a microscopic diffusion tensor **D**. This assumption has been widely employed in the recent development of multidimensional diffusion MR (see ref.^4^ for a recent review and the references therein), which employs general gradient waveforms for diffusion sensitization. The level of diffusion-sensitivity is fully captured by a measurement tensor^5^ **B**, yielding the signal attenuation

1 $$S={e}^{-{\rm{tr}}({\bf{D}}{\bf{B}})}$$

for the microdomain. When some residual anisotropy is present upon the inherent signal averaging over the sample (or voxel in image acquisitions), a series of diffusion signals can be acquired with rotated versions of the same gradient waveforms. Upon averaging such signals, one obtains the orientationally-averaged signal, which is devoid of any macroscopic (ensemble) anisotropy, i.e., the anisotropy of the orientation distribution function of the microdomains.

As demonstrated in ref.^4^, there is a close resemblance between the mathematics involved in this problem with that in multidimensional solid-state NMR spectroscopy^6^. In the latter, the local structure is described by the chemical shift tensor, and a measurement tensor can be introduced, which is determined by the orientation of the main magnetic field and manipulations of the sample orientation within it^7–11^.

Our interest in this article is the averaged signal obtained by repeating a given measurement protocol in all orientations, which is relevant for both diffusion and solid-state MR applications. Our results permit a more general analysis and modeling when the orientationally-averaged signal has been measured, as the common condition on axisymmetry is relaxed. The problem of determining the set of optimal diffusion gradient orientations for computing the orientationally-averaged signal is addressed elsewhere^12^. Here, we extend the existing literature^3,13,14^ by providing explicit expressions for the orientational averages to accommodate measurement and/or structure tensors that are *not* axisymmetric.

With **R** denoting an arbitrary rotation matrix (${\bf{R}}{{\bf{R}}}^{\top }={{\bf{R}}}^{\top }{\bf{R}}={\bf{I}}$), the complete set of such measurements is spanned by the expression ${\bf{R}}{\bf{B}}{{\bf{R}}}^{\top }$, hence yielding the orientationally-averaged signal as

2a $$\bar{S}={\langle {e}^{-{\rm{tr}}({\bf{D}}{\bf{R}}{\bf{B}}{{\bf{R}}}^{\top })}\rangle }_{{\bf{R}}},$$

where the average is over the three dimensional special orthogonal group SO(3), i.e., the space of all rotations. Since the matrix trace operation is invariant under cyclic permutation of the product in its argument, this is equivalent to the expression

2b $$\bar{S}={\langle {e}^{-{\rm{tr}}({{\bf{R}}}^{\top }{\bf{D}}{\bf{R}}{\bf{B}})}\rangle }_{{\bf{R}}},$$

which demonstrates the utility of such an averaging over rotated protocols: The information is the same as that which would be obtained by a single measurement, had the specimen consisted of microdomains with the same diffusivity (**D**) distributed uniformly in orientation. For solids, such a specimen is obtained by grinding the material into a powder, eliminating any nonuniformity in the orientational dispersion (macroscopic, global, or ensemble anisotropy) the bulk specimen might have had. Hence we use the terms *powder*-averaged and *orientationally*-averaged interchangeably, given the latter effectively achieves the same result.

An alternative interpretation of the average in (2b) can be realized when one considers the Laplace transform of a function which takes matrix arguments^15^, in our case a tensor distribution *p*(**D**),

3 $${ {\mathcal L} }_{p({\bf{D}})}({\bf{B}})={\int }_{{{\rm{Sym}}}_{3}}\,{e}^{-{\rm{tr}}({\bf{D}}{\bf{B}})}\,p({\bf{D}})\,{\rm{d}}{\bf{D}},$$

where the integration is performed over Sym_3_, the space of all symmetric 3 × 3 matrices, and for those matrices ${\bf{B}}\in {{\rm{Sym}}}_{3}$ where the integral converges. With ${\bar{{\mathscr{P}}}}_{3}$ denoting the set of all positive semi-definite matrices in Sym_3_, the applications of interest in this work will be when all the matrices ${\bf{D}}\in {\overline{{\mathscr{P}}}}_{3}$. This means that $p({\bf{D}})=0$ if **D** is not in ${\overline{{\mathscr{P}}}}_{3}$, so that the integration in (3) can be performed over ${\overline{{\mathscr{P}}}}_{3}$ rather than Sym_3_. ${ {\mathcal L} }_{p({\bf{D}})}({\bf{B}})$ will then exist for all ${\bf{B}}\in {\overline{{\mathscr{P}}}}_{3}$.

In (3), the integration measure is ${\rm{d}}{\bf{D}}={\rm{\Pi }}\,{\rm{d}}{D}_{ij}$ with $1\le i\le j\le 3$. Interestingly, when $p({\bf{D}})$ is such that it describes an ensemble of tensors consisting of copies of a given tensor $\hat{{\bf{D}}}$, rotated to lie in all possible orientations, the above expression is the same average as (2b). In this scenario $p({\bf{D}})=0$ unless ${\bf{D}}\sim \hat{{\bf{D}}}$, i.e, ${\bf{D}}={{\bf{R}}}^{\top }\hat{{\bf{D}}}{\bf{R}}$ for some **R** in SO(3). When ${\bf{D}}\sim \hat{{\bf{D}}}$ we require $p({\bf{D}})=p(\hat{{\bf{D}}})$, which gives us (2b) (with $\hat{{\bf{D}}}$ replaced by **D**). In other words, the orientationally-averaged signal that we are evaluating in this work is nothing but the Laplace transform of the distribution $p({\bf{D}})$ describing a uniform ensemble of rotated copies of a given tensor. Parallels can be drawn with previous works^16–18^ that have employed parametric Wishart distributions (or its generalizations) for representing the detected MR signal; in these studies, the resulting expression for the Laplace transform is borrowed from the mathematics literature^19^. However, the Laplace transform of the distribution we are tackling in this work (Eq. (2b)) for a general **D** is not available to our knowledge.

Note that throughout the article, the phrase “general tensor” refers to a tensor whose ellipsoidal representation is not axisymmetric. Thus, a general tensor **T** is still to be understood as being real-valued and having the index symmetry ${T}_{ij}={T}_{ji}$. Given that the matrices **D** and **B** are real and symmetric, and that the averages (2) are insensitive to individual rotations of these matrices, we are free to consider their diagonal forms, possibly in different bases

4 $${\bf{D}}=(\begin{array}{ccc}a & 0 & 0\\ 0 & b & 0\\ 0 & 0 & c\end{array}),\,{\bf{B}}=(\begin{array}{ccc}d & 0 & 0\\ 0 & e & 0\\ 0 & 0 & f\end{array}).$$

It turns out that the average signal (2) can take a particularly simple form if repeated eigenvalues occur in at least one of the matrices **D** and **B**. With an ellipsoidal representation of these matrices in mind, we refer to these cases as *axisymmetric* or *isotropic*, depending on whether two or all eigenvalues coincide, respectively. The rank of the matrices appear to have no further significance for the simplicity of the calculation, except for rank-1 being a special case of axisymmetry.

In the following sections, we consider various combinations of symmetries of **D** and **B** in evaluating the average (2). Keeping in mind that the roles of **D** and **B** can be interchanged without changing the average signal, the relevant cases are

1. **D** general, **B** isotropic,
2. **D** and **B** axisymmetric, **B** rank 1,
3. **D** and **B** axisymmetric,
4. **D** general, **B** axisymmetric,
5. **D** and **B** general.

## Results

Here, we give the resulting expressions for the average signal (2) in the cases listed above, with **D** and **B** given as in Eq. (4). All derivations are provided in Supplementary Section I, where $\bar{S}$ is first derived for the case (4) of a general **D** and axisymmetric **B**, from which more specialized cases (1–3) are deduced, while the most general case (5) is taken up last.

### D general, B isotropic

This is the case where all eigenvalues (*a*, *b*, *c*) of **D** are possibly different, while **B** is proportional to identity with $d=e=f$. One finds,

5 $$\bar{S}={e}^{-d(a+b+c)}={e}^{-d{\rm{tr}}({\bf{D}})}.$$

Indeed, when ${\bf{B}}=d{\bf{I}}$, ${\rm{tr}}({\bf{D}}{\bf{R}}{\bf{B}}{{\bf{R}}}^{\top })={\rm{tr}}(d{\bf{D}})$ and hence the average in Eq. (2) has no effect.

### **D** and **B** axisymmetric, **B** rank 1

Here, two eigenvalues of **D** coincide, which we choose as $b=c$, while **B** is rank-1 with $e=f=0$. This is a widely-utilized case in diffusion MR^1,3,20–24^. The result follows as,

6 $$\bar{S}=\tfrac{\sqrt{\pi }{e}^{-cd}}{2}\tfrac{\mathrm{erf}(\sqrt{d(a-c)})}{\sqrt{d(a-c)}}=\tfrac{\sqrt{\pi }{e}^{-cd}}{2}\tfrac{\mathrm{erfi}(\sqrt{d(c-a)})}{\sqrt{d(c-a)}},$$

where ${\rm{erf}}(\,\cdot \,)$ is the error function and ${\rm{erfi}}(\,\cdot \,)$ is the imaginary error function; ${\rm{erfi}}(x)={\rm{erf}}(ix)/i$. One can choose either of the formulas in (6), but depending on the sign of $d(a-c)$, one expression will have real arguments, and the other imaginary. For instance, with $d > 0$, $d(a-c) > 0$ if **D** is prolate and $d(a-c) < 0$ if **D** is oblate. It is assumed that $d\ne 0$ and $a\ne c$, as this implies that one of the matrices is isotropic, but this case can of course also be included here by continuity arguments.

### **D** and **B** axisymmetric

When both matrices have axisymmetry, with $b=c$ and $e=f$, we have^13^

7 $$\bar{S}=\tfrac{\sqrt{\pi }{e}^{-cd-f(a+c)}}{2}\tfrac{\mathrm{erf}(\sqrt{(a-c)\,(d-f)})}{\sqrt{(a-c)\,(d-f)}}=\tfrac{\sqrt{\pi }{e}^{-cd-f(a+c)}}{2}\tfrac{\mathrm{erfi}(\sqrt{(c-a)\,(d-f)})}{\sqrt{(c-a)\,(d-f)}}\mathrm{.}$$

With similar remarks as in the previous case, any of the two forms in (7) can be chosen, and if moreover $a=c$ or $d=f$, one gets the first (isotropic) case above. The first form has real arguments when both **D** and **B** are either prolate or oblate, while the second form may be preferable when one matrix is prolate and the other oblate.

### **D** general, **B** axisymmetric

For the almost-general case where the only condition is axisymmetry of **B** with $e=f$, the result is given in four alternative forms by

8a $$\bar{S}=\{\begin{array}{ll}{e}^{-f{\rm{tr}}({\bf{D}})}\tfrac{\sqrt{\pi }{e}^{(f-d)c}}{2}\,\sum _{n=0}^{\infty }\,\tfrac{{[(c-a)(d-f)]}^{n}}{(n+1/2)!}\,{}_{2}F_{1}(\tfrac{1}{2},-\,n;\,\mathrm{1;}\,\tfrac{a-b}{a-c}), & a\ne c\\ \tfrac{\sqrt{\pi }{e}^{-ad-(a+b)f}}{2}\tfrac{\mathrm{erf}(\sqrt{(b-a)\,(d-f)})}{\sqrt{(b-a)\,(d-f)}}=\tfrac{\sqrt{\pi }{e}^{-ad-(a+b)f}}{2}\tfrac{\mathrm{erfi}(\sqrt{(a-b)\,(d-f)})}{\sqrt{(a-b)\,(d-f)}}, & a=c\,({\rm{case}}\,\mathrm{3)}\end{array}$$

8b $$\begin{array}{rcl} & = & {e}^{-f{\rm{tr}}({\bf{D}})}{e}^{(f-d)c}\,\sum _{n=0}^{\infty }\,\tfrac{{[(a-b)(d-f)]}^{n}}{\mathrm{(2}n+\mathrm{1)}n!}\,{}_{1}F_{1}(n+\mathrm{1;}\,n+\tfrac{3}{2};(c-a)\,(d-f))\end{array}$$

8c $$\begin{array}{rcl} & = & {e}^{-f{\rm{tr}}({\bf{D}})}\tfrac{\sqrt{\pi }{e}^{(f-d)c}}{2}\,\sum _{n=0}^{\infty }\,\tfrac{{[(c-a)(d-f)]}^{n}}{(n+1/\mathrm{2)!}}\,{}_{2}F_{2}(\tfrac{1}{2},n+1;1,n+\tfrac{3}{2};(a-b)\,(d-f))\end{array}$$

8d $$\begin{array}{rcl} & = & {e}^{-f{\rm{tr}}({\bf{D}})}\tfrac{\sqrt{\pi }{e}^{(f-d)c}}{2}\,\sum _{n=0}^{\infty }\,\tfrac{{[(a-b)(d-f)]}^{2n}}{{16}^{n}(2n+\tfrac{1}{2})!}(\begin{array}{c}2n\\ n\end{array})\,{}_{1}F_{1}(2n+1;2n+\tfrac{3}{2};\tfrac{1}{2}(f-d)\,(a+b-2c))\mathrm{.}\end{array}$$

Here, the symbol _*i*_*F*_*j*_$(\,\cdot \,)$ stands for a hypergeometric function, in particular with _1_*F*_1_$(\,\cdot \,)$ being the confluent hypergeometric function. Equations (8a–c) were derived by a rather direct approach; see Supplementary Section I.A. The fourth expression (8d), which is perhaps the most interesting for $\bar{S}$ when one matrix is axisymmetric, is derived from the general expression, i.e, the case accounted for next. As discussed below, it can lead to very efficient numerical evaluation.

The expressions above feature hypergeometric functions. However, it is possible to express them using more familiar functions. For example, by using a scheme^25^ that involves term by term comparison with the confluent hypergeometric function’s asymptotic behaviour^26^, we were able to write Eq. (8b) in the following form:

9 $$\begin{array}{rcl}\bar{S} & = & {e}^{-f{\rm{tr}}({\bf{D}})}\,\sum _{k=0}^{\infty }\,\tfrac{\mathrm{(2}k)!}{{(k!)}^{2}{4}^{k}}\,{[(a-b)(d-f)]}^{k}\\  &  & \times \,\{{e}^{(f-d)c}\,\sum _{n=1}^{k}\,\tfrac{1}{{\mathrm{[2(}a-c)(d-f)]}^{n}}\,\sum _{j=0}^{k-n}\,\tfrac{{(-\mathrm{1)}}^{j+1}\mathrm{(2}n+2j-\mathrm{1)!!}}{(k-n-j)!(n+j\mathrm{)!(2}j+\mathrm{1)!!}}\\  &  & +\,{e}^{(f-d)a}\tfrac{\sqrt{\pi }\,\mathrm{erfi}(\sqrt{(a-c)(d-f)})}{2\sqrt{(a-c)\,(d-f)}}\,\sum _{n=0}^{k}\,\tfrac{\mathrm{(2}n-\mathrm{1)!!}}{n!(k-n)!}\tfrac{1}{{\mathrm{[2(}a-c)(d-f)]}^{n}}\},\end{array}$$

where $(2n+1)!!$ denotes the product of all odd numbers from 1 up to $(2n+1)$. Similarly, Eq. (8a) can be expressed via more familiar functions, using Eq. (11).

We also note that in the literature the signal for this case is reduced to a one-dimensional integral wherein the integrand is given by a complete elliptic integral^6,14^.

### **D** and **B** general

In the general case where neither matrix (necessarily) has any degenerate eigenvalue, the result can be expressed as

10a $$\bar{S}={e}^{-\frac{1}{2}(a+b)(e+f)-cd}\,\sum _{0\leqslant m\leqslant k < \infty }\,{q}_{mk}{Y}_{mk},$$

where

10b $${q}_{mk}=\{\begin{array}{ll}(\begin{array}{ll}\tfrac{{((c-a)(e-f))}^{k}}{{4}^{k}{((k/\mathrm{2)!)}}^{2}}, & m=0\,{\rm{and}}\,k\,{\rm{even}}\\ \mathrm{0,} & {\rm{otherwise}}\end{array}, & a=b\\ \tfrac{1}{{2}^{k}}{(c-a)}^{k-m}\,\sum _{j=\lfloor \tfrac{m+1}{2}\rfloor }^{k\mathrm{/2}}\,{I}_{k-j}(\tfrac{(e-f)\,(a-b)}{2}) & \\ \times {(a-b)}^{m-j}{(e-f)}^{k-j}\tfrac{1}{j!(k-2j)!}(\begin{array}{c}2j\\ m\end{array}), & a+b-2c=0\\ \tfrac{{(c-a)}^{k-m}}{{2}^{k}(k-m)!}\,\sum _{j=0}^{k/2}\,{I}_{k-j}(\tfrac{(e-f)\,(a-b)}{2})\tfrac{{(a+b-2c)}^{m-2j}{(a-b)}^{j}{(e-f)}^{k-j}}{j!} & \\ \times {}_{2}\tilde{F}_{1}(m-k,-\,2j;\,1+m-2j,\tfrac{a+b-2c}{a-b}), & \,\,{\rm{otherwise}}\end{array}$$

and there are three alternatives for *Y*_*mk*_ as follows:

10c $${Y}_{mk}^{\mathrm{(1)}}=\{\begin{array}{ll}\tfrac{\sqrt{\pi }{4}^{-m}}{2}(\begin{array}{c}2m\\ m\end{array})\,\sum _{n=0}^{\infty }\,\tfrac{{(\tfrac{1}{2}(c-a)\mathrm{(2}d-e-f))}^{n}(k+n)!\,{}_{2}F_{1}(m+\tfrac{1}{2},-n;m+1;\tfrac{a-b}{a-c})}{n!(k+n+1\,/\,\mathrm{2)!}}, & a\ne c\\ \tfrac{\sqrt{\pi }{4}^{-m}}{2}(\begin{array}{c}2m\\ m\end{array})\,\tfrac{k!}{(k+\mathrm{1/2})!} & \\ {}_{3}F_{2}(1,k+1,m+\frac{1}{2};k+\,\frac{3}{2},\,m+1;\frac{1}{2}(a-b)\,\mathrm{(2}d-e-f)), & a=c\end{array}$$

10d $${Y}_{mk}^{(2)}=\frac{\sqrt{\pi }{4}^{-m}}{2}\,\sum _{n=0}^{\infty }\,\tfrac{{(\tfrac{1}{2}(a-b)(2d-e-f))}^{n}(k+n)!(\begin{array}{c}2(n+m)\\ n+m\end{array})\,{}_{1}F_{1}(k+n+1;k+n+\tfrac{3}{2};\tfrac{1}{2}(c-a)\,(2d-e-f))}{{4}^{n}n!(k+n+1\,/\,2)!},$$

10e $${Y}_{mk}^{(3)}=\frac{\sqrt{\pi }{4}^{-m}}{2}\,(\begin{array}{c}2m\\ m\end{array})\,\sum _{n=0}^{\infty }\,\tfrac{{(\tfrac{1}{2}(c-a)(2d-e-f))}^{n}(k+n)!\,{}_{2}F_{2}(m+\tfrac{1}{2},k+n+1;m+1,k+n+\tfrac{3}{2};\tfrac{1}{2}(a-b)\,(2d-e-f))}{n!(k+n+1\,/\,2)!}.$$

The coefficients *q*_*mk*_ contain *I*_*m*_$(\,\cdot \,)$, i.e., the modified Bessel function of the first kind for various orders *m*, and the regularized hypergeometric function ${}_{2}\tilde{F}_{1}(\,\cdot \,)$. Also note that the floor function $\lfloor \,\cdot \,\,\,\rfloor$ indicates the largest integer smaller than or equal to its argument.

It is straightforward to check that $\bar{S}$ as given by Eq. (10) is invariant under the rescaling ${\bf{D}}\to \lambda {\bf{D}}$, ${\bf{B}}\to \tfrac{1}{\lambda }{\bf{B}}$ for nonzero *λ*, verifying an obvious invariance it should have due to its definition (2). Implied by that definition, $\bar{S}$ must also be unaffected by permutations of $\{a,b,c\}$ and of $\{d,e,f\}$, as well as swapping $\{a,b,c\}\leftrightarrow \{d,e,f\}$. This is not at all evident in the expansions above; in fact the ordering of eigenvalues can have drastic effects on the numerical behaviour of the series, even though the eventual sum does not change. Given **D** and **B**, and their eigenvalues, the remaining task is thus to assign them (in some order) to *a*, *b*, *c* and *d*, *e*, *f* in such a way that the series can be approximated well with only a few terms; see the next section.

## Numerical Behaviour

In this section we give some comments and guidelines on how to evaluate $\bar{S}$ via the series in Eqs (8) and (10). The numerical behaviour of the series given in Eqs (8) and (10) varies drastically depending on how the eigenvalues of **D** and **B** are ordered (i.e., which eigenvalue is named *a*, *b*, and so on). In the formulas in Eq. (10), there is also the option of interchanging **D** and **B**. The discussion here revolves around an example employing Eq. (8), but the guidelines apply to the general case of Eq. (10) as well, albeit more involved.

When **B** is axisymmetric ($e=f$), *f* and *d* are fixed in Eq. (8). On the other hand, in these formulas, which all give the same result, one is free to permute $\{a,b,c\}$. Although this does not affect the answer, it affects the number of terms needed to get a good approximation. Hence, given the three eigenvalues of **D**, one wants to assign them to *a*, *b*, *c* in a clever way, as well as choose the most efficient formula.

Looking at the expansions (8), while there appears no obvious “best” choice for assigning a given set of eigenvalues, it is still wise to try and (*i*) keep the series expansion parameter (e.g., $(a-b)\,(d-f)$ in Eq. 8d) small, and (*ii*) avoid alternating signs, since an expansion where all terms have the same sign cannot suffer from cancellation effects. For instance, it is worth paying attention to the magnitude and sign of $(c-a)\,(d-f)$ and _2_*F*_1_$(\tfrac{1}{2},-\,n;1;\tfrac{a-b}{a-c})$ in Eq. (8a), and so on for the rest. Therefore, even though a deep analysis of the properties of hypergeometric functions is beyond the scope of this work, it is useful to note the following as a guideline for their sign and magnitude. With $n\in {\bf{N}}$, the functions _2_*F*_1_$(\frac{1}{2},-\,n;1;x) > 0$ are positive and decreasing functions of $x < 0$, and happen to be polynomials. On the other hand, _2_*F*_2_$(\frac{1}{2},n+1;1,n+\frac{3}{2};x) > 0$ are positive and increasing functions of $x\ge 0$. Finally, the functions _1_*F*_1_$(n+1;n+\frac{3}{2};x) > 0$ are positive and increasing for all $x\in {\bf{R}}$. (See Supplementary Section II for some elaboration on this).

**Figure 1:**
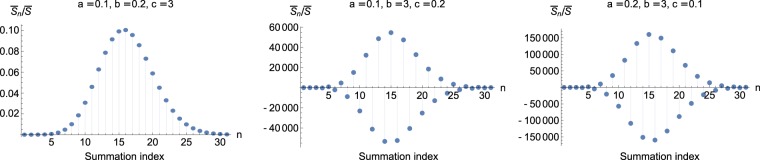
The first terms in the series in Eq. (8a), which uses the hypergeometric function _2_*F*_1_. The terms ${\bar{S}}_{n}$ are normalized by $\bar{S}$ so that they add up to 1. **B** has eigenvalues $d=6$, $e=f=0.5$, while **D** has eigenvalues $0.1,0.2,3$. Depending on how the latter are assigned to *a*, *b* and *c*, the trend of the series expansion varies. Displayed are three (instead of six) distinct choices, because each term is invariant under the change $a\leftrightarrow b$, which is not obvious from Eq. (8a), but can be shown using Eq. (19).

**Figure 2:**
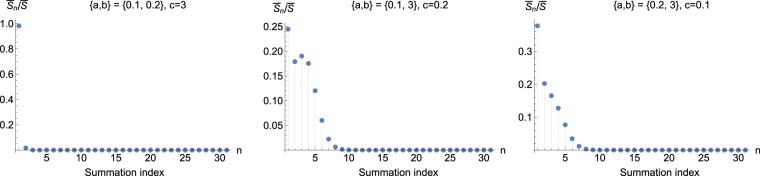
The first terms in the series in Eq. (8d), which uses the hypergeometric function _1_*F*_1_. The terms ${\bar{S}}_{n}$ are normalized by $\bar{S}$ so that they add up to 1. **B** has eigenvalues $d=6$, $e=f=0.5$, while **D** has eigenvalues $0.1,0.2,3$. Depending on how the latter are assigned to *a*, *b* and *c*, the trend of the series expansion varies. Displayed are three (instead of six) distinct choices, since individual terms of Eq. (8d) are manifestly invariant under swapping $a\leftrightarrow b$.

In Figs 1 and 2, the contributions of individual terms in the series are displayed, with the index of the terms running on the horizontal axis. The figures correspond, respectively, to the alternative forms (8a) and (8d), which were chosen as examples to illustrate clearly that term by term each series exhibits different behaviours depending on the allocation of the eigenvalues to the parameters *a*, *b*, *c*. For the particular case presented in the figures, the matrix **D** has eigenvalues 0.1, 0.2, 3 and the matrix **B** has eigenvalues 6, 0.5, 0.5, for which $\bar{S}\approx 0.019175$, and all the terms are normalized by $\bar{S}$ so that their sum is 1.

The first alternative (8a) has the property that its individual terms are invariant under the change $a\leftrightarrow b$, which can be shown to follow from relation (11). Therefore, out of the six possible assignments between the elements of the sets $\{a,b,c\}$ and $\{0.1,0.2,3\}$ only three are distinct. These are what is depicted in Fig. 1. It is seen clearly that for these parameters this alternative does not afford a series expansion that can be truncated after the first few terms for a usable result. The terms making the most significant contribution to the sum are not even at the beginning, but in this example rather around term number 15. Furthermore, the two latter choices exhibit terms of alternating sign and magnitudes of about 10^5^ times the sum itself.

Figure 2 depicts all three distinct choices for the alternative expression (8d). We see a very desirable feature here. When the largest value is assigned to *c*, the terms are seen to converge quickly (note that the expression is invariant under the exchange $$a\leftrightarrow b$$). With this choice (e.g., $$a=0.2,b=0.1,c=3$$) we have _1_*F*_1_$$(2n+1;2n+3/2;$$$$(f-d)(a+b-2c)/2)$$ = _1_*F*_1_$$(2n+1;2n+3/2;15.675)$$ which is bounded with respect to *n*. In fact, it follows from termwise comparison in the defining series for _1_*F*_1_ (see Supplementary Section II) that _1_*F*_1_$$\mathrm{(2}n+\mathrm{1,}\,2n+\mathrm{3/2;}\,\mathrm{15.675)}$$ $$\le $$_1_*F*_1_$$(2n+3/2;2n+3/2;15.675)={e}^{15.675}$$. We also see that only even powers $$(a-b)\,(d-f)=0.55$$ enter, resulting in all terms of the series being positive; no cancellation occurs. This choice leads to a sum that converges so quickly that $$\bar{S}$$ is approximated within 2% by only the first term, while the first two terms attain an error less than 0.01%. In addition, it should also be noted that for moderate values of *n*, _1_*F*_1_$$(n+1;n+3/2;x)$$ are explicitly expressible in elementary functions and that for _1_*F*_1_ there are effective recursive relations at hand (see Supplementary Section II).

### Swapping *a* and *b*

By construction, all series are unaffected by permutations of *a*, *b*, *c* (although it affects the numerical behaviour) but in the series (8a), also the individual terms are unaffected by the change $a\leftrightarrow b$. This follows from the relation

11 $${}_{2}F_{1}(\tfrac{1}{2},-\,n;1;z)={(1-z)}^{n/2}{P}_{n}(\frac{2-z}{2\sqrt{1-z}}),$$

where ${P}_{n}(\,\cdot \,)$ is the *n*th order Legendre polynomial. It is then readily verified that ${(a-c)}^{n}$ _2_*F*_1_$(\tfrac{1}{2},-\,n;1;\tfrac{a-b}{a-c})$ and ${(b-c)}^{n}$ _2_*F*_1_$(\tfrac{1}{2},-\,n;1;\tfrac{b-a}{b-c})$ are indeed equal. The terms in the series (8d) are also unaffected by the change $a\leftrightarrow b$, but this is immediate from the expression.

## Applications

The results in this paper are the exact expressions given in (8–10), which extends the earlier formulas (5–7), together with the asymptotic behaviour given by Theorem 1. These series can give exact answers in more general models where axisymmetry is not insisted upon. However, to further motivate applicability of our findings, we discuss and suggest several possible situations where the new results provided in this work are used.

We have presented explicit formulas for the orientationally-averaged signal $\bar{S}$ in Eq. (2) for general (symmetric) matrices **D** and **B**. These formulas complement the well-known cases 1, 2, and 3, i.e., the formulas in Eqs (5–7), where **D** and **B** have various symmetries. However, even when one matrix, say **B**, is rank-1, the signal expression for a general **D** (Eq. 8) is believed to be new. Eq. (10) raises the question of the usefulness of case 5, in which both measurement and diffusion tensors are general. We argue that this solution is indeed useful, for example, when a powdered specimen whose local structure is characterized by a general diffusion tensor is examined. In MRI, the imaging gradients lead to a rank-3 measurement tensor even when a standard Stejskal-Tanner sequence is employed. Moreover, since for a general **D**, even using a rank-1 measurement tensor $${\bf{B}}=(\begin{array}{ccc}d & 0 & 0\\ 0 & 0 & 0\\ 0 & 0 & 0\end{array})$$, the knowledge of $\bar{S}(d)$ for sufficiently many (at least 3) *d* determines **D**. Below, we also discuss power-laws and asymptotic behaviour of the signal in a more general setting.

From a practical standpoint, many “independent” measurements are necessary in order to mitigate noise-related effects. With an axisymmetric measurement tensor **B**, the space of measurements is two dimensional (two degrees of freedom in **B**), while in the general case the space of measurements is three dimensional. *Loosely speaking*, this means that in the latter case, there are more “independent” measurement tensors available close to the origin of this space (or any other point common to both spaces, for that matter). Since measurements close to the origin (i.e., **B** tensors with small eigenvalues) produce higher signal value, this is favorable from a signal-to-noise ratio perspective. On the theoretical side, however, there are situations where a general **B** tensor actually is crucial in determining the diffusivity properties of the specimen; see subsection below on the estimation of **D** from the series expansion of $\bar{S}$.

### Remarks on the white-matter signal at large diffusion-weighting

Recently, the orientationally-averaged signal in white-matter regions of the brain was observed to follow the power-law $\bar{S}\propto {d}^{-\mathrm{1/2}}$ at large *d* when $e=f=0$, e.g., in traditional Stejskal-Tanner measurements^27,28^. Because such decay is predicted for vanishing transverse diffusivity, these results have been interpreted to justify the “stick” model of axons^21,29,30^ ($a > b=c=0$) while also suggesting that the signal from the extra-axonal space disappears at large diffusion-weighting. In a recent article^24^, we showed that such a decay is indeed expected for one-dimensional curvilinear diffusion observed via narrow pulses. On the other hand, in acquisitions involving wide pulses, the curvature of the fibers has to be limited in order for $\bar{S}\propto {d}^{-\mathrm{1/2}}$ behaviour to emerge. We also noted that the $\bar{S}\propto {d}^{-\mathrm{1/2}}$ dependence is valid for an intermediate range of diffusion weightings as the true asymptotics of the signal decay is governed by a steeper decay^24^.

Here, based on these findings, we consider a rank-1 diffusion tensor for representing intra-axonal diffusion, which is capable of reproducing the intermediate $\bar{S}(d,\,\mathrm{0,}\,\mathrm{0)}\propto {d}^{-\mathrm{1/2}}$ dependence for measurement tensors of the form $${\bf{B}}=(\begin{array}{ccc}d & 0 & 0\\ 0 & 0 & 0\\ 0 & 0 & 0\end{array})$$. For rank-2, axisymmetric measurement tensors, $${\bf{B}}=(\begin{array}{ccc}d & 0 & 0\\ 0 & d & 0\\ 0 & 0 & 0\end{array})$$, the orientationally-averaged decay is characterized by the power-law ${\bar{S}}_{a}(d,d,0)\propto {d}^{-1}$ with *a* being the single nonzero eigenvalue of **D**, which can be shown to follow from Eq. (6) upon interchanging **D** and **B**. For non-axisymmetric, rank-2 measurement tensors, the signal, $\bar{S}(d,e,0)$ with $d > e$ obeys the relationship

12 $${\bar{S}}_{a}(d,d,0)\le {\bar{S}}_{a}(d,e,0)\le {\bar{S}}_{a}(e,e,0).$$

Now, consider the case in which the measurement is tuned by “inflating” the **B**-matrix while preserving its shape, i.e., varying *d* while keeping *e*/*d* fixed. By this we mean that we calculate (and fix) the ratio $\lambda =e/d$ so that ${\bar{S}}_{a}(d,d,0)\le {\bar{S}}_{a}(d,e,0)$ = ${\bar{S}}_{a}(d,\lambda d,0)\le {\bar{S}}_{a}(\lambda d,\lambda d,0)$. Then, varying only *d*, both the lower and upper bounds indicated in the above expression decay according to ${\bar{S}}_{a}\propto {d}^{-1}$. For ${\bar{S}}_{a}(d,d,0)$, this is clear, and for the upper bound we have ${\bar{S}}_{a}(e,e,0)={\bar{S}}_{a}(\lambda d,\lambda d,0)={\bar{S}}_{\lambda a}(d,d,0)\propto {d}^{-1}$ (since *λ* is constant and non-zero). To satisfy these inequalities, it must thus also be that ${\bar{S}}_{a}(d,e,0)\propto {d}^{-1}$, which establishes the decay of the orientationally-averaged signal decay obtained via *general* rank-2 (**B** matrices). Thus, an alternative validation of the stick model could be performed by acquiring data using planar encoding, in which case the expected decay of the orientationally averaged signal would be characterized by a decay $\propto \,{d}^{-1}$ regardless of whether or not the **B** matrix is axisymmetric.

### Observation of the (non)axisymmetry of local diffusion tensors

Here, we focus our attention to rank-1 measurement tensors, $${\bf{B}}=d(\begin{array}{ccc}1 & 0 & 0\\ 0 & 0 & 0\\ 0 & 0 & 0\end{array})$$, e.g., obtained using the Stejskal-Tanner sequence, and investigate to what extent **D** is determined when $\bar{S}={\bar{S}}_{{\bf{D}}}(d)$ is known for some values *d*. Specifically, we ask the question: Is it possible to distinguish an axisymmetric **D** from a non-axisymmetric **D**?

**Figure 3:**
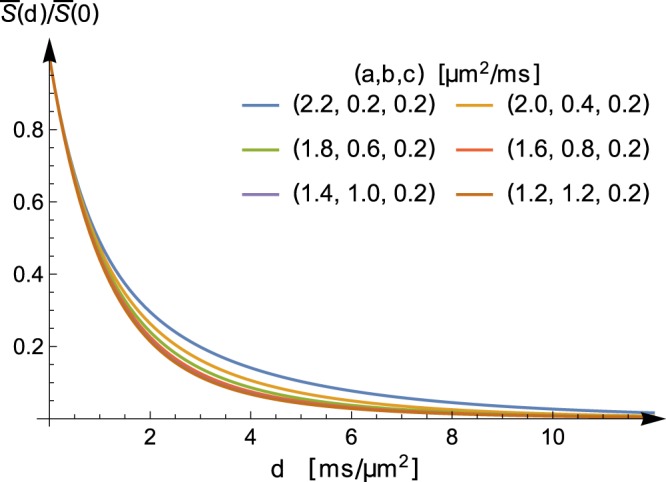
Given the rank-1 measurement tensor **B**, with eigenvalues $(d,0,0)$, the signal ${\bar{S}}_{{{\bf{D}}}_{i}}(d)$ is plotted as a function of *d* for six different diffusion tensors ${{\bf{D}}}_{1},{{\bf{D}}}_{2},\ldots {{\bf{D}}}_{6}$. These diffusion tensors all have the same trace, and their eigenvalues $(a,b,c)$ are in order ${{\bf{D}}}_{1}:(2.2,0.2,0.2)$ *μ*m^2^/ms, ${{\bf{D}}}_{2}:(2.0,0.4,0.2)$ *μ*m^2^/ms, ${{\bf{D}}}_{3}:(1.8,0.6,0.2)$ *μ*m^2^/ms, ${{\bf{D}}}_{4}:(1.6,0.8,0.2)$ *μ*m^2^/ms, ${{\bf{D}}}_{5}:(1.4,1.0,0.2)$ *μ*m^2^/ms, ${{\bf{D}}}_{6}:(1.2,1.2,0.2)$ *μ*m^2^/ms.

The answer to this question is yes; in fact, it is not so hard to see that if ${\bar{S}}_{{{\bf{D}}}_{1}}(d)$ and ${\bar{S}}_{{{\bf{D}}}_{2}}(d)$ are equal for all $d\geqslant 0$, then **D**_1_ and **D**_2_ must be equal—see for instance the remark near Eq. (17) in the next section. (Also recall that we identify the matrices according to their eigenvalues; two matrices are “equal” for the purposes of this article if one can be rotated into the other.) As an illustration, starting with a rank-1 measurement tensor $${\bf{B}}=d(\begin{array}{ccc}1 & 0 & 0\\ 0 & 0 & 0\\ 0 & 0 & 0\end{array})$$, Fig. 3 shows the signal $\bar{S}$ as a function of $d$ for six different diffusion tensors ${{\bf{D}}}_{1},{{\bf{D}}}_{2},\ldots {{\bf{D}}}_{6}$. In this example, all tensors **D**_*i*_, $i=1,2,\ldots 6$ have the same trace yielding the same behaviour near the origin, and their common smallest eigenvalue leads to the same large *b* behaviour. By varying the remaining two eigenvalues, (whose values are found in Fig. 3) while keeping their sum constant, different curves ${\bar{S}}_{{{\bf{D}}}_{i}}(d)$ are produced. The six curves shown in Fig. 3 are samples from a family (indexed by the diffusion tensor **D**) of curves characterized by their initial and asymptotic behaviour. It is interesting to note that no axisymmetric diffusion tensor **D** other than **D**_1_ and **D**_6_ can produce a curve in this family. In this context, the ‘same asymptotic behaviour’ refers to the same exponential decay as $d\to \infty$, and this is given by the smallest eigenvalue of the diffusion tensor. The initial behaviour, i.e., $\bar{S^{\prime} }\mathrm{(0)}$ is given by the trace of the diffusivity, and it is immediate to see that an axisymmetric tensor where the smallest eigenvalue is 0.2 *μ*m^2^/ms and where the trace is 2.6 *μ*m^2^/ms has to have the eigenvalues of either **D**_1_ or **D**_6_.

In the remaining part of this section, we consider the intermediate *d* regime alluded to in the previous section. For a general rank-3 diffusion tensor $${\bf{D}}=(\begin{array}{ccc}a & 0 & 0\\ 0 & b & 0\\ 0 & 0 & c\end{array})$$, the falloff is exponential, which can be inferred from the expression (for $a\ge b\ge c > 0$)

13 $${\bar{S}}_{a,a,c}(d)\le {\bar{S}}_{a,b,c}(d)\le {\bar{S}}_{a,c,c}(d)$$

since both the left- and the right-hand-sides of the above expression decay exponentially fast; see (6). Consequently, a reliable inference cannot be made in the large *d* regime in typical acquisitions due to limited SNR. The only exception is when $c=0$, i.e., when **D** is of rank 2. In this case, the arguments from the preceding section can be employed by interchanging the matrices **D** and **B**, i.e.,

14 $${\bar{S}}_{a,a,0}(d)\le {\bar{S}}_{a,b,0}(d)\le {\bar{S}}_{b,b,0}(d),$$

where, ${\bar{S}}_{a,a,0}(d)={(2ad)}^{-1}$ for large *d*. Hence we expect (at least when $a > b$)

15 $$\frac{1}{2ad}\le {\bar{S}}_{a,b,0}(d)\le \frac{1}{2bd}\,{\rm{for}}\,{\rm{large}}\,d.$$

This is indeed the case, as the following theorem shows.

#### **Theorem 1**.

*Suppose that the diffusion tensor* **D** *has eigenvalues a*, *b*, 0 *where a*, *b* > 0, *and that the measurement tensor* **B** *has eigenvalues d*, 0, 0. *Denoting the corresponding powder average with* ${\bar{S}}_{a,b,0}(d)$, *it then holds that*

$$\mathop{\mathrm{lim}}\limits_{d\to \infty }\,d{\bar{S}}_{a,b,0}(d)=\frac{1}{2\sqrt{ab}}.$$

#### *Proof*.

See Supplementary Section V.$\square$

**Figure 4:**
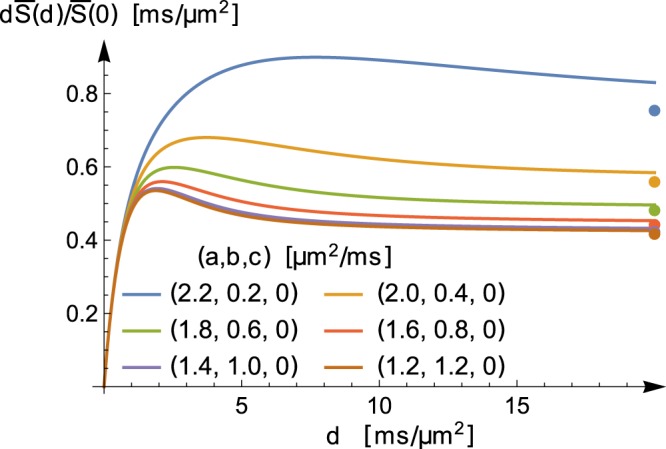
Given the rank-1 measurement tensor **B**, with eigenvalues $(d,0,0)$, the product $d{\bar{S}}_{{{\bf{D}}}_{i}}(d)$ is plotted as a function of *d* for six different diffusion tensors ${{\bf{D}}}_{1},{{\bf{D}}}_{2},\ldots {{\bf{D}}}_{6}$. These diffusion tensors all have the same trace, and their eigenvalues $(a,b,c)$ are in order ${{\bf{D}}}_{1}:(2.2,0.2,0)$ *μ*m^2^/ms, ${{\bf{D}}}_{2}:(2.0,0.4,0)$ *μ*m^2^/ms, ${{\bf{D}}}_{3}:(1.8,0.6,0)$ *μ*m^2^/ms, ${{\bf{D}}}_{4}:(1.6,0.8,0)$ *μ*m^2^/ms, ${{\bf{D}}}_{5}:(1.4,1.0,0)$ *μ*m^2^/ms, ${{\bf{D}}}_{6}:(1.2,1.2,0)$ *μ*m^2^/ms. Using Theorem 1, ${\mathrm{lim}}_{d\to \infty }\,d{\bar{S}}_{{{\bf{D}}}_{i}}(d)$ are known, and the corresponding values are shown with dots. The discrepancy between the curves and the corresponding dots are due to the finite values of *d*.

This limiting behaviour is illustrated in Fig. 4 where, starting with a measurement tensor $${\bf{B}}=d(\begin{array}{ccc}1 & 0 & 0\\ 0 & 0 & 0\\ 0 & 0 & 0\end{array})$$, plots of the signal ${\bar{S}}_{{{\bf{D}}}_{i}}(d)$ are shown for six different diffusion tensors **D**_*i*_, $i=1,2\ldots 6$. The eigenvalues $(a,b,c)$ of these diffusion tensors are ${{\bf{D}}}_{1}:(2.2,0.2,0)$ *μ*m^2^/ms, ${{\bf{D}}}_{2}:(2.0,0.4,0)$ *μ*m^2^/ms, ${{\bf{D}}}_{3}:(1.8,0.6,0)$ *μ*m^2^/ms, ${{\bf{D}}}_{4}:(1.6,0.8,0)$ *μ*m^2^/ms, ${{\bf{D}}}_{5}:(1.4,1.0,0)$ *μ*m^2^/ms, ${{\bf{D}}}_{6}:(1.2,1.2,0)$ *μ*m^2^/ms. Note that all diffusion tensors have the same trace. For each such tensor, the conditions of Theorem 1 are met, and the limits (as $d\to \infty$) $\tfrac{1}{2\sqrt{ab}}$ can be calculated. These limits are shown with dots in Fig. 4. The discrepancy between the curves and the corresponding dots are due to the finite values of *d*.

Hence, for large *d*, ${\bar{S}}_{a,b,0}(d)\sim {[\kappa (a,b)d]}^{-1}$ with $\kappa (a,b)=2\sqrt{ab}$, which evaluates to ${\rm{tr}}({\bf{D}})$ only for $a=b$. On the other hand, the small *d* behaviour of the average signal is always ${\bar{S}}_{{\bf{D}}}\approx 1-\frac{d}{3}{\rm{tr}}({\bf{D}})$. Thus, ${\rm{tr}}({\bf{D}})$ can, in principle, be determined from the initial decay of the average signal. Any mismatch between this estimate and the estimate of $\kappa (a,b)$ from the power-law decay of the signal can thus be attributed to transverse anisotropy of **D**.

The above inference is a clear example to how the derived expressions involving general tensors can be utilized to gain new insight into the characterization of the local structure.

### Estimation of D from the power series expansion of $\bar{{\boldsymbol{S}}}$

One of the main results of this paper consists of the series in Eq. (10), which make it possible to calculate the powder average $\bar{S}$ for general matrices **D** and **B** without numerical integration or sampling in SO(3). As the corresponding formula (8) for the case when one of the matrices, **B**, is axisymmetric is simpler, a relevant question is whether the use of a general **B** is of any help in determining **D** from a set of measurements. This question immediately splits into two; namely if there is an advantage with a general **B** in principle, and also in practice. For instance, for any fixed $\tilde{{\bf{B}}}$, and regarding $\bar{S}$ as a function of ${\bf{B}}=\lambda \tilde{{\bf{B}}}$, the asymptotics of $\bar{S}$ for large *λ* may be of primary importance in principle, but in practice the actual values of $\bar{S}$ in that regime may be so small that noise and measurement errors make them impossible to use for determining **D**.

To address the question above, we start by considering a general **B** and then the special case when **B** is axisymmetric. Since any axisymmetric matrix **B** is the sum of an isotropic matrix and a rank-one matrix, and since the effect on $\bar{S}$ of an isotropic matrix is trivial, c.f. Eq. (5), it is sufficient to consider rank-one matrices **B**.

So, with a general **D**, we consider a family of **B**-matrices given by ${\bf{B}}=\lambda \tilde{{\bf{B}}}$, where $$\tilde{{\bf{B}}}=(\begin{array}{ccc}d & 0 & 0\\ 0 & e & 0\\ 0 & 0 & f\end{array})$$ is a fixed matrix, and *λ* is a scalar strength parameter. For fixed **D** and $\tilde{{\bf{B}}}$, the orientationally-averaged signal ${\bar{S}}_{{\bf{D}}}({\bf{B}})={\bar{S}}_{{\bf{D}}}(\lambda \tilde{{\bf{B}}})=\bar{S}(\lambda )$ is a function of *λ*, and we seek the dependence for small *λ*. Consider therefore the expansion ${\bar{S}}_{{\bf{D}}}(\lambda \tilde{{\bf{B}}})=1+{c}_{1}({\bf{D}},\tilde{{\bf{B}}})\lambda +{c}_{2}({\bf{D}},\tilde{{\bf{B}}}){\lambda }^{2}+{c}_{3}({\bf{D}},\tilde{{\bf{B}}}){\lambda }^{3}+{\mathscr{O}}({\lambda }^{4})$, $\lambda \to 0$. The coefficients ${c}_{1},{c}_{2},{c}_{3}$ can be estimated from the knowledge of ${\{{\bar{S}}_{{\bf{D}},\tilde{{\bf{B}}}}({\lambda }_{i})\}}_{i}$ for a suitable set of (small) {*λ*_*i*_}_*i*_. Up to third order (see Supplementary Section III) the coefficients *c*_*k*_ are

16 $$\begin{array}{rcl}{c}_{1} & = & -\frac{1}{3}{\rm{tr}}({\bf{D}}){\rm{tr}}(\tilde{{\bf{B}}})\\ {c}_{2} & = & \frac{1}{30}(2{{\rm{tr}}}^{2}({\bf{D}}){{\rm{tr}}}^{2}(\tilde{{\bf{B}}})+3{\rm{tr}}({{\bf{D}}}^{2}){\rm{tr}}({\tilde{{\bf{B}}}}^{2})-{{\rm{tr}}}^{2}({\bf{D}}){\rm{tr}}({\tilde{{\bf{B}}}}^{2})-{\rm{tr}}({{\bf{D}}}^{2}){{\rm{tr}}}^{2}(\tilde{{\bf{B}}}))\\ {c}_{3} & = & \frac{1}{630}[{\rm{tr}}({{\bf{D}}}^{3})(\,-\,36{\rm{tr}}({\tilde{{\bf{B}}}}^{3})+36{\rm{tr}}({\tilde{{\bf{B}}}}^{2}){\rm{tr}}(\tilde{{\bf{B}}})-8{{\rm{tr}}}^{3}(\tilde{{\bf{B}}}))\\  &  & +\,{\rm{tr}}({{\bf{D}}}^{2}){\rm{tr}}({\bf{D}})(36{\rm{tr}}({\tilde{{\bf{B}}}}^{3})-57{\rm{tr}}({\tilde{{\bf{B}}}}^{2}){\rm{tr}}(\tilde{{\bf{B}}})+15{{\rm{tr}}}^{3}(\tilde{{\bf{B}}}))\\  &  & +\,{{\rm{tr}}}^{3}({\bf{D}})(\,-\,8{\rm{tr}}({\tilde{{\bf{B}}}}^{3})+15{\rm{tr}}({\tilde{{\bf{B}}}}^{2}){\rm{tr}}(\tilde{{\bf{B}}})-8{{\rm{tr}}}^{3}(\tilde{{\bf{B}}}))].\end{array}$$

For a generic matrix $\tilde{{\bf{B}}}$ (with the isotropic choice being the singular exception), knowledge of these three coefficients suffice to determine the matrix **D**: For ease of illustration, consider the special case wherein $\tilde{{\bf{B}}}$ is rank-1 (i.e., $e=f=0$) yielding the simplified coefficients

17 $$\begin{array}{rcl}{c}_{1} & = & -\frac{d}{3}{\rm{tr}}({\bf{D}})\\ {c}_{2} & = & \frac{{d}^{2}}{30}({{\rm{tr}}}^{2}({\bf{D}})+2{\rm{tr}}({{\bf{D}}}^{2}))\\ {c}_{3} & = & -\frac{{d}^{3}}{630}({{\rm{tr}}}^{3}({\bf{D}})+6{\rm{tr}}({{\bf{D}}}^{2}){\rm{tr}}({\bf{D}})+8{\rm{tr}}({{\bf{D}}}^{3})).\end{array}$$

We see that this is a non-degenerate system that gives ${\rm{tr}}({\bf{D}}),{\rm{tr}}({{\bf{D}}}^{2}),{\rm{tr}}({{\bf{D}}}^{3})$ in terms of ${c}_{1},{c}_{2},{c}_{3}$, and since (the eigenvalues of) **D** is determined from these three traces, *c*_1_, *c*_2_ and *c*_3_ determine **D**.

Returning to a general $\tilde{{\bf{B}}}$, a convenient point of view is to consider $\tilde{{\bf{B}}}$ as decomposed into two parts (c.f., Supplementary Section I) and write, $$\tilde{{\bf{B}}}=(\begin{array}{ccc}\delta  & 0 & 0\\ 0 & \varepsilon  & 0\\ 0 & 0 & 0\end{array})+f{\bf{I}}$$, where $\delta =d-f,\varepsilon =e-f$. Then the average signal (2) factors into two, each factor calculable from $$\tilde{{\bf{B}}}=(\begin{array}{ccc}\delta  & 0 & 0\\ 0 & \varepsilon  & 0\\ 0 & 0 & 0\end{array})$$ and $\tilde{{\bf{B}}}=f{\bf{I}}$ separately. We find that when $$\tilde{{\bf{B}}}=(\begin{array}{ccc}\delta  & 0 & 0\\ 0 & \varepsilon  & 0\\ 0 & 0 & 0\end{array})$$, the expansion coefficients (16) attain the simplified form

18 $$\begin{array}{rcl}{c}_{1} & = & -\frac{1}{3}(\delta +\varepsilon ){\rm{tr}}({\bf{D}})\\ {c}_{2} & = & \frac{1}{30}[({\delta }^{2}+{\varepsilon }^{2})({{\rm{tr}}}^{2}({\bf{D}})+2{\rm{tr}}({{\bf{D}}}^{2}))+\delta \varepsilon (4{{\rm{tr}}}^{2}({\bf{D}})-2{\rm{tr}}({{\bf{D}}}^{2}))]\\ {c}_{3} & = & -\frac{1}{630}[({\delta }^{3}+{\varepsilon }^{3})\,({{\rm{tr}}}^{3}({\bf{D}})+6{\rm{tr}}({\bf{D}}){\rm{tr}}({{\bf{D}}}^{2})+8{\rm{tr}}({{\bf{D}}}^{3}))\\  &  & +\,({\delta }^{2}\varepsilon +\delta {\varepsilon }^{2})\,(9{{\rm{tr}}}^{3}({\bf{D}})+12{\rm{tr}}({\bf{D}}){\rm{tr}}({{\bf{D}}}^{2})-12{\rm{tr}}({{\bf{D}}}^{3}))],\end{array}$$

while an isotropic $\tilde{{\bf{B}}}=f{\bf{I}}$ on the other hand yields (renaming the coefficients)

19 $${g}_{1}=-\,f\,{\rm{tr}}({\bf{D}}),\,{g}_{2}=\frac{{f}^{2}}{2}{{\rm{tr}}}^{2}({\bf{D}}),\,{g}_{3}=-\,\frac{{f}^{3}}{6}{{\rm{tr}}}^{3}({\bf{D}}).$$

The resulting signal expansion is the product $$\bar{S}(\lambda )=[1+{c}_{1}\lambda +{c}_{2}{\lambda }^{2}+{c}_{3}{\lambda }^{3}+{\mathscr{O}}({\lambda }^{4})]$$ $$[1+{g}_{1}\lambda +{g}_{2}{\lambda }^{2}+$$$${g}_{3}{\lambda }^{3}+{\mathscr{O}}({\lambda }^{4})]$$. Note how the isotropic part is not all that helpful: It attenuates the signal without being sensitive to anything other than $${\rm{tr}}({\bf{D}})$$.

When using a general $\tilde{{\bf{B}}}$ as described above, Eq. (18) tells us that **D** cannot be determined from *c*_1_ and *c*_2_ alone, even if we are using the extra degree of freedom in **B** by varying *δ* and $\varepsilon$. This is so since *c*_1_ and *c*_2_ contain information only on two invariants (${\rm{tr}}({\bf{D}})$ and ${\rm{tr}}({{\bf{D}}}^{2})$), which is not sufficient to determine **D**, which has three eigenvalues. That being said, in practice, varying *δ* and $\varepsilon$ may be a way of getting “independent” measurements to reduce the influence of noise, i.e., increase SNR and therefore provide more reliable estimates of the coefficients *c*_*k*_.

Let us also mention that in the situation described by Eq. (18) where the combined freedom of *δ* and $\varepsilon$ offers no extra information, one can still vary these to test the assumption that the specimen is indeed described by a single diffusivity matrix **D**. Namely, for different values of *δ* and $\varepsilon$, one should always get the same estimated matrix **D**.

### Standard model of white-matter with a general diffusion tensor for the extracellular compartment

The extra degree of freedom provided by the parameters *δ* and $\varepsilon$ may be crucial in certain relevant situations. In principle, such situations can be addressed by involving higher order coefficients ${c}_{4},{c}_{5},\ldots$ but reliably estimating them would get increasingly difficult, and using ${c}_{1},{c}_{2},{c}_{3}$ for various *δ* and $\varepsilon$ values is more robust. We give the following example, which could be relevant for simplified models of white-matter microstructure.

Suppose that our specimen is a mixture of two different substances, in unknown proportions, where one of the substances is (from a diffusivity perspective) a “stick”. Thus, in proportions *p* and $1-p$, with $0\leqslant p\leqslant 1$, we have the unknown diffusivity matrices $${\bf{D}}\sim (\begin{array}{ccc}a & 0 & 0\\ 0 & b & 0\\ 0 & 0 & c\end{array})$$ and $$\tilde{{\bf{D}}}\sim (\begin{array}{ccc}q & 0 & 0\\ 0 & 0 & 0\\ 0 & 0 & 0\end{array})$$. This model is similar to the commonly employed white-matter model (sometimes referred to as the “standard model”^31^), but differs from it in two ways: (i) we ignore the isotropic compartment, whose contribution to the orientationally-averaged signal is simply an exponential (see (5)), (ii) the contribution from the extracellular matrix is given by a general diffusion tensor, which is not necessarily axially-symmetric.

In this model, we therefore have five unknowns to determine; three invariants of **D** together with *p* and *q*. Since ${\rm{tr}}({\tilde{{\bf{D}}}}^{k})={q}^{k},k=1,2,3,\ldots$, Eq. (18) now becomes

20 $$\begin{array}{rcl}(\delta +\varepsilon )(p\,{\rm{tr}}({\bf{D}})+\mathrm{(1}-p)q) & = & -3{c}_{1}(\delta ,\varepsilon )\\ ({\delta }^{2}+{\varepsilon }^{2})[p({{\rm{tr}}}^{2}({\bf{D}})+2{\rm{tr}}({{\bf{D}}}^{2}))+\mathrm{(1}-p\mathrm{)3}{q}^{2}] &  & \\ +\delta \varepsilon [p(4{{\rm{tr}}}^{2}({\bf{D}})-2{\rm{tr}}({{\bf{D}}}^{2}))+\mathrm{(1}-p\mathrm{)2}{q}^{2}] & = & 30{c}_{2}(\delta ,\varepsilon )\\ ({\delta }^{3}+{\varepsilon }^{3})[p({{\rm{tr}}}^{3}({\bf{D}})+6{\rm{tr}}({\bf{D}}){\rm{tr}}({{\bf{D}}}^{2})+8{\rm{tr}}({{\bf{D}}}^{3}))+\mathrm{(1}-p\mathrm{)15}{q}^{3}] &  & \\ +({\delta }^{2}\varepsilon +\delta {\varepsilon }^{2})[p(9{{\rm{tr}}}^{3}({\bf{D}})+12{\rm{tr}}({\bf{D}}){\rm{tr}}({{\bf{D}}}^{2})-12{\rm{tr}}({{\bf{D}}}^{3}))+\mathrm{(1}-p\mathrm{)9}{q}^{3}] & = & -630{c}_{3}(\delta ,\varepsilon \mathrm{).}\end{array}$$

By varying *δ* and/or $\varepsilon$, these equations determine $p,q,{\bf{D}}$ “almost uniquely”, in the sense that there are (rare) situations where there are two sets of acceptable solutions to Eq. (20). However, if one also adds the measurements with an isotropic measurement tensor, this possible ambiguity goes away. (See Supplementary Section IV for details.) Thus, it is possible to obtain all 5 unknowns of a multi-compartment white-matter model from the first three terms of the power-series representation of the orientationally-averaged signal.

## Discussion and Conclusions

Our elaborations on the numerical behaviour of the main results (8) and (10) were restricted to the case where one matrix is axisymmetric, corresponding to Eq. (8). In the more general case corresponding to Eq. (10), the same guidelines apply regarding how the ordering of the eigenvalues affects the numerical behaviour, with some additional caveats. First of all, since no eigenvalues necessarily coincide, the number of possible distinguishable orderings increases, by a factor of 6 in particular. Also, not only one has to choose between the three alternatives (10c–e) by the same guidelines that apply to the alternatives (8a–d), but also the number of coefficients *q*_*mk*_ has to be specified.

A more brute-force approach to calculate the orientational average (2) is to set up the necessary integrations in the space of rotations and perform them by some discretization scheme. However, such an approach would require the integrand ${e}^{-{\rm{tr}}({\bf{D}}{\bf{R}}{\bf{B}}{{\bf{R}}}^{\top })}$ to be relatively well-behaved in the integration domain in order to be accurate, which is not always the case. For instance, when the matrices **D** and **B** are very similar to each other, and with one eigenvalue dominating the others, the integrand is (virtually) zero for most rotations **R**, with most of the contribution stemming from a small subset. In such cases, a discretization of the average is not reliable.

In this work, we represented local diffusion by employing a diffusion tensor along with the generalization^32^ of the Stejskal-Tanner formula^33^ for the signal contribution of each microdomain. The underlying assumption is that diffusion in each and every microdomain is unrestricted. This assumption^16^ has been the building block of not only the microstructure models mentioned above, but also the techniques^5,34–36^ developed within the multidimensional diffusion MRI framework. Introduction of the confinement tensor concept^37,38^ provides a viable direction that could achieve the same by accounting for the possible restricted character of the microdomains. This is the subject of future work.

Another limitation of the present work is the assumption that there is no variation in the size of the microdomains making up the complex environment—the same assumption employed in many of the microstructure models. Previous studies^39,40^ suggest the complexity of accounting for such variations in different contexts. We intend to address this issue in the future.

In conclusion, we studied the orientationally-averaged magnetic resonance signal by extending the existing expressions to cases involving general tensors with no axisymmetry. This was accomplished by evaluating a challenging average (Eq. (2)), or equivalently the integral in (3) for the special class of *p*(**D**) distributions considered in this article. Although the results are given as sums of infinitely many terms, we showed that with certain arrangements of the parameters, obtaining very accurate estimates is possible by retaining a few terms in the series. These developments led to a number of interesting inferences on the properties of the signal decay curve as well as estimation of relevant parameters from the signal.

The findings presented in this work could be useful in many contexts in which the the expression (2) (or (3)) emerges. For example, though we employed the nomenclature of diffusion MR in this paper, our findings are applicable to solid-state NMR spectroscopy as well due to the mathematical similarities of the two fields.

## Supplementary information

Supplementary information

## References

[1] Callaghan PT, Jolley KW, Lelievre J (1979). Diffusion of water in the endosperm tissue of wheat grains as studied by pulsed field gradient nuclear magnetic resonance. Biophys J.

[2] Mitra PP, Sen PN (1992). Effects of microgeometry and surface relaxation on NMR pulsed-field-gradient experiments: Simple pore geometries. Phys Rev B.

[3] Joabsson F, Nydén M, Linse P, Söderman O (1997). Pulsed field gradient NMR studies of translational diffusion in cylindrical surfactant aggregates. J Phys Chem B.

[4] Topgaard D (2017). Multidimensional diffusion MRI. J Magn Reson.

[5] Westin CF (2014). Measurement tensors in diffusion MRI: generalizing the concept of diffusion encoding. Lect Notes Comput Sc.

[6] Schmidt-Rohr K, Spiess HW (1994). Multidimensional Solid-State NMR and Polymers.

[7] Andrew ER, Bradbury A, Eades RG (1959). Removal of dipolar broadening of nuclear magnetic resonance spectra of solids by specimen rotation. Nature.

[8] Szeverenyi NM, Bax A, Maciel GE (1985). Magic-angle hopping as an alternative to magic-angle spinning for solid state NMR. J Magn Reson.

[9] Bax A, Szeverenyl NM, Maciel GE (1983). Chemical shift anisotropy in powdered solids studied by 2D FT NMR with flipping of the spinning axis. J Magn Reson.

[10] Ziegler RC, Wind RA, Maciel GE (1988). The stop-and-go spinning technique in MAS experiment. J Magn Reson.

[11] Frydman L (1992). Variable-angle correlation spectroscopy in solid-state nuclear magnetic resonance. J Chem Phys.

[12] Szczepankiewicz, F., Westin, C.-F. & Knutsson, H. A measurement weighting scheme for optimal powder average estimation. In *Proc Intl Soc Mag Reson Med*, vol. 26, 3345 (2017).

[13] Eriksson S, Lasič S, Nilsson M, Westin C-F, Topgaard D (2015). NMR diffusion-encoding with axial symmetry and variable anisotropy: Distinguishing between prolate and oblate microscopic diffusion tensors with unknown orientation distribution. J Chem Phys.

[14] Bloembergen N, Rowland TJ (1953). On the nuclear magnetic resonance in metals and alloys. Acta Metallurgica.

[15] Herz CS (1955). Bessel functions of matrix argument. Ann Math.

[16] Jian B, Vemuri BC, Özarslan E, Carney PR, Mareci TH (2007). A novel tensor distribution model for the diffusion-weighted MR signal. NeuroImage.

[17] Scherrer B (2016). Characterizing brain tissue by assessment of the distribution of anisotropic microstructural environments in diffusion-compartment imaging (DIAMOND). Magn Reson Med.

[18] Shakya, S., Batool, N., Özarslan, E. & Knutsson, H. Multi-fiber reconstruction using probabilistic mixture models for diffusion MRI examinations of the brain. In Schultz, T., Özarslan, E. & Hotz, I. (eds) *Modeling*, *Analysis*, *and Visualization of Anisotropy*, 283–308 (Springer International Publishing, Cham, 2017).

[19] Letac G, Massam H (1998). Quadratic and inverse regressions for Wishart distributions. Ann Stat.

[20] Yablonskiy DA (2002). Quantitative *in vivo* assessment of lung microstructure at the alveolar level with hyperpolarized ^3^He diffusion MRI. Proc Natl Acad Sci USA.

[21] Kroenke CD, Ackerman JJH, Yablonskiy DA (2004). On the nature of the NAA diffusion attenuated MR signal in the central nervous system. Magn Reson Med.

[22] Anderson AW (2005). Measurement of fiber orientation distributions using high angular resolution diffusion imaging. Magn Reson Med.

[23] Kaden E, Kelm ND, Carson RP, Does MD, Alexander DC (2016). Multi-compartment microscopic diffusion imaging. NeuroImage.

[24] Özarslan E, Yolcu C, Herberthson M, Knutsson H, Westin C-F (2018). Influence of the size and curvedness of neural projections on the orientationally averaged diffusion mr signal. Front Phys.

[25] Özarslan E, Shepherd TM, Vemuri BC, Blackband SJ, Mareci TH (2006). Resolution of complex tissue microarchitecture using the diffusion orientation transform (DOT). NeuroImage.

[26] Abramowitz M, Stegun IA (1977). Handbook of Mathematical Functions: With Formulas, Graphs, and Mathematical Tables.

[27] McKinnon ET, Jensen JH, Glenn GR, Helpern JA (2017). Dependence on b-value of the direction-averaged diffusion-weighted imaging signal in brain. Magn Reson Imaging.

[28] Novikov DS, Kiselev VG, Jespersen SN (2018). On modeling. Magn Reson Med.

[29] Behrens TEJ (2003). Characterization and propagation of uncertainty in diffusion-weighted MR imaging. Magn Reson Med.

[30] Zhang H, Schneider T, Wheeler-Kingshott CA, Alexander DC (2012). NODDI: practical *in vivo* neurite orientation dispersion and density imaging of the human brain. NeuroImage.

[31] Novikov, D. S., Fieremans, E., Jespersen, S. N. & Kiselev, V. G. Quantifying brain microstructure with diffusion mri: Theory and parameter estimation. *NMR Biomed* e3998, 10.1002/nbm.3998 (2018). 10.1002/nbm.3998 PMC6481929 30321478

[32] Karlicek RF, Lowe IJ (1980). A modified pulsed gradient technique for measuring diffusion in the presence of large background gradients. J Magn Reson.

[33] Stejskal EO (1965). Use of spin echoes in a pulsed magnetic-field gradient to study anisotropic, restricted diffusion and flow. J Chem Phys.

[34] Lasič S, Szczepankiewicz F, Eriksson S, Nilsson M, Topgaard D (2014). Microanisotropy imaging: quantification of microscopic diffusion anisotropy and orientational order parameter by diffusion mri with magic-angle spinning of the q-vector. Frontiers in Physics.

[35] Szczepankiewicz F (2015). Quantification of microscopic diffusion anisotropy disentangles effects of orientation dispersion from microstructure: applications in healthy volunteers and in brain tumors. NeuroImage.

[36] Westin CF (2016). Q-space trajectory imaging for multidimensional diffusion MRI of the human brain. NeuroImage.

[37] Yolcu C, Memiç M, Şimşek K, Westin CF, Özarslan E (2016). NMR signal for particles diffusing under potentials: From path integrals and numerical methods to a model of diffusion anisotropy. Phys Rev E.

[38] Özarslan E, Yolcu C, Herberthson M, Westin C-F, Knutsson H (2017). Effective potential for magnetic resonance measurements of restricted diffusion. Front Phys.

[39] Yablonskiy DA, Bretthorst GL, Ackerman JJH (2003). Statistical model for diffusion attenuated MR signal. Magn Reson Med.

[40] Özarslan E, Shemesh N, Koay CG, Cohen Y, Basser PJ (2011). Nuclear magnetic resonance characterization of general compartment size distributions. New J Phys.

