# Supplementary material for: Orientationally-averaged diffusion-attenuated magnetic resonance signal for locally-anisotropic diffusion
Source: Sci Rep. 2019 Mar 20;9:4899. doi: 10.1038/s41598-019-41317-8 (PMC6426978; doi:10.1038/s41598-019-41317-8)
Supplement: Supplementary file 1 — Supplementary information [file 41598_2019_41317_MOESM1_ESM.pdf]

Orientationally-averaged diffusion-attenuated magnetic resonance signal for locally-anisotropic diffusion: Supplementary information

Magnus Herberthson,[1, a)] Cem Yolcu,[2] Hans Knutsson,[2] Carl-Fredrik Westin,[2, 3] and Evren Özarslan[2, 4]
[1)] *Dept. of Mathematics, Linköping University, Linköping, Sweden*
[2)] *Dept. of Biomedical Engineering, Linköping University, Linköping, Sweden*
[3)] *Laboratory for Mathematics in Imaging, Dept. of Radiology, Brigham and Women's Hospital, Harvard Medical School, Boston, MA, USA*
[4)] *Center for Medical Image Science and Visualization, Linköping University, Linköping, Sweden*

I. CALCULATION OF THE INTEGRALS

In this appendix, we derive the formulas (5–10). After some setup and general remarks, we start by deriving Eq. (8), i.e., the case when one matrix is axisymmetric, in I A. From this the formulas (5–7) follow as special cases.

The result (8) for the case involving one axisymmetric matrix is in itself a special case of the general result (10) where both $\mathbf{D}$ and $\mathbf{B}$ are general. However, this situation is more involved and is addressed in I B.

Note that as Eqs. (2) stand, it is enough that either $\mathbf{D}$ or $\mathbf{B}$ is symmetric, as $\mathrm{tr}(\mathbf{R}^\intercal\mathbf{D}\mathbf{R}\mathbf{B}) = 0$ if $\mathbf{D}$ is symmetric and $\mathbf{B}$ is antisymmetric, or vice versa. Also, by the physical set up, it is natural to consider all the eigenvalues of $\mathbf{D}$ and $\mathbf{B}$ to be non-negative, but this is not insisted on in the calculations. Moreover, throughout the article, fractional factorials are defined via the Gamma function, i.e., $z! = \Gamma(z+1)$.

We start by rewriting (in some basis which is not important)

$$\mathbf{D} = c\mathbf{I} + \left(\begin{smallmatrix}\alpha & 0 & 0\\ 0 & \beta & 0\\ 0 & 0 & 0\end{smallmatrix}\right), \text{ with}$$
$$\alpha = a - c\ ,$$
$$\beta = b - c\ ,$$

and similarly (in perhaps a different basis)

$$\mathbf{B} = f\mathbf{I} + \left(\begin{smallmatrix}\delta & 0 & 0\\ 0 & \epsilon & 0\\ 0 & 0 & 0\end{smallmatrix}\right), \text{ with}$$
$$\delta = d - f\ ,$$
$$\epsilon = e - f\ .$$

Thus, $B = f\mathbf{I} + \delta \boldsymbol{u}\boldsymbol{u}^\intercal + \epsilon \boldsymbol{v}\boldsymbol{v}^\intercal$ for some pair of orthogonal unit vectors $\boldsymbol{u}, \boldsymbol{v}$. Using this, we find that

$$\mathrm{tr}(\mathbf{D}\mathbf{R}\mathbf{B}\mathbf{R}^\intercal) = c(d+e) + f(a+b) - cf + \mathrm{tr}\left[\left(\begin{smallmatrix}\alpha & 0 & 0\\ 0 & \beta & 0\\ 0 & 0 & 0\end{smallmatrix}\right)\mathbf{R}(\delta\boldsymbol{u}\boldsymbol{u}^\intercal + \epsilon\boldsymbol{v}\boldsymbol{v}^\intercal)\mathbf{R}^\intercal\right]$$

Noting that $\mathrm{tr}(\mathbf{D}\boldsymbol{x}\boldsymbol{x}^\intercal) = \boldsymbol{x}^\intercal\mathbf{D}\boldsymbol{x}$, and defining

$$Q_0 = c(d+e) + f(a+b) - cf\ , \tag{S1}$$

the original expression (2) for the powder-averaged signal becomes

$$\bar{S} = e^{-Q_0}\left\langle e^{-\delta(\mathbf{R}\boldsymbol{u})^\intercal\left(\begin{smallmatrix}\alpha & 0 & 0\\ 0 & \beta & 0\\ 0 & 0 & 0\end{smallmatrix}\right)\mathbf{R}\boldsymbol{u} - \epsilon(\mathbf{R}\boldsymbol{v})^\intercal\left(\begin{smallmatrix}\alpha & 0 & 0\\ 0 & \beta & 0\\ 0 & 0 & 0\end{smallmatrix}\right)\mathbf{R}\boldsymbol{v}}\right\rangle_{\mathbf{R}\in\mathrm{SO}(3)}\ . \tag{S2}$$

The mean over rotation matrices $\mathbf{R}$ will be taken in the following way (this is related to the Hopf fibration[1,2] of S^3, with S^3 being the double cover of SO(3)).

[a)] Electronic address: magnus.herberthson@liu.se

Given the two orthogonal unit vectors $\boldsymbol{u}$ and $\boldsymbol{v}$, a rotation matrix $\mathbf{R}$ is determined by the (mutually orthogonal) images $\mathbf{R}\boldsymbol{u}$ and $\mathbf{R}\boldsymbol{v}$. Thus $\boldsymbol{u}$ will run over all unit vectors and for each instance, $\boldsymbol{v}$ will run over all possible directions orthogonal to it. Using standard spherical coordinates, we let

$$\boldsymbol{u} = \begin{pmatrix} \sin\theta\cos\phi \\ \sin\theta\sin\phi \\ \cos\theta \end{pmatrix}, \; \boldsymbol{v} = \begin{pmatrix} -\sin\phi \\ \cos\phi \\ 0 \end{pmatrix}, \tag{S3}$$

and let $\mathbf{R}_{\boldsymbol{u}}(\mu)$ denote a rotation matrix which rotates an angle μ around $\boldsymbol{u}$. From the exponent in Eq. (S2), we put

$$\begin{aligned} Q_1 &= \delta(\mathbf{R}_{\boldsymbol{u}}(\mu)\boldsymbol{u})^{\mathsf{T}} \left(\begin{smallmatrix} \alpha & 0 & 0 \\ 0 & \beta & 0 \\ 0 & 0 & 0 \end{smallmatrix}\right) \mathbf{R}_{\boldsymbol{u}}(\mu)\boldsymbol{u} = \delta \boldsymbol{u}^{\mathsf{T}} \left(\begin{smallmatrix} \alpha & 0 & 0 \\ 0 & \beta & 0 \\ 0 & 0 & 0 \end{smallmatrix}\right) \boldsymbol{u} \\ &= \delta \sin^2\theta(\alpha\cos^2\phi + \beta\sin^2\phi) = \delta(\alpha + (\beta-\alpha)\sin^2\phi)\sin^2\theta, \end{aligned} \tag{S4}$$

which is independent of μ, and

$$Q_2 = \epsilon\,(\mathbf{R}_{\boldsymbol{u}}(\mu)\boldsymbol{v})^{\mathsf{T}} \left(\begin{smallmatrix} \alpha & 0 & 0 \\ 0 & \beta & 0 \\ 0 & 0 & 0 \end{smallmatrix}\right) \mathbf{R}_{\boldsymbol{u}}(\mu)\boldsymbol{v}, \tag{S5}$$

where it easy to check that

$$R_{\boldsymbol{u}}(\mu)\boldsymbol{v} = R_{\boldsymbol{u}}(\mu)\begin{pmatrix} -\sin\phi \\ \cos\phi \\ 0 \end{pmatrix} = \begin{pmatrix} -\sin\mu\cos\phi\cos\theta - \cos\mu\sin\phi \\ \cos\mu\cos\phi - \sin\mu\sin\phi\cos\theta \\ \sin\mu\sin\theta \end{pmatrix}.$$

Now the average (S2) can be rewritten as,

$$\bar{S} = \frac{e^{-Q_0}}{(4\pi)(2\pi)} \int_0^{2\pi}\int_0^{\pi} e^{-Q_1} \int_0^{2\pi} e^{-Q_2}\, \mathrm{d}\mu \sin\theta\, \mathrm{d}\theta\, \mathrm{d}\phi\ . \tag{S6}$$

Evaluation of the angular average in the above expression in the general case is provided in I B, while the resulting expressions were given in *Results*.

However, we start by looking at the special case with one matrix, say $\mathbf{B}$, being axisymmetric. One can then arrange its eigenvalues so that $\epsilon = 0$, which yields $Q_2 = 0$ and means that the angular average (S6) takes the form

$$\begin{aligned} \bar{S} &= \frac{e^{-Q_0}}{4\pi} \int_0^{2\pi}\int_0^{\pi} e^{-Q_1} \sin\theta\, \mathrm{d}\theta\, \mathrm{d}\phi \\ &= \frac{e^{-Q_0}}{4\pi} \int_0^{2\pi}\int_0^{\pi} e^{-\delta(\alpha+(\beta-\alpha)\sin^2\phi)\sin^2\theta} \sin\theta\, \mathrm{d}\theta\, \mathrm{d}\phi\ , \end{aligned} \tag{S7}$$

as a result of the μ integral dropping out since Q_2 is the only place where μ-dependence might have been present. Moreover, one of the integrations can readily be reduced by using either

$$\frac{1}{2\pi}\int_0^{2\pi} e^{-a\sin^2\phi}\, \mathrm{d}\phi = e^{-a/2} I_0(\tfrac{a}{2})$$

or

$$\frac{1}{\pi}\int_0^{\pi} e^{-a\sin^2\theta} \sin\theta\, \mathrm{d}\theta = \frac{e^{-a}}{\sqrt{a\pi}} \mathrm{erfi}\left(\sqrt{a}\right).$$

Here I_0 is a modified Bessel function of the first kind (with order 0) and erfi is the imaginary error function, $\mathrm{erfi}(x) = \mathrm{erf}(ix)/i$. Hence, with one of the matrices $\mathbf{D}$ and $\mathbf{B}$ axisymmetric, it is easy to write the orientational average (2) as definite integral of one variable. However, aiming for the result (8), where we have expressed (S7) as a series rather than an integral, we proceed as follows.

A. D general, B axisymmetric

Suppose that one matrix, say $\mathbf{B}$, is axisymmetric, and that we let the two equal eigenvalues be $e = f$, so that $\epsilon = 0$. As noted in above, $\bar{S}$ is then given by Eq. (S7). Putting for brevity $\zeta = \delta\alpha$ and $\gamma = \delta(\beta - \alpha)$, the integrand has the form

$$e^{-(\zeta+\gamma\sin^2\phi)\sin^2\theta} = \sum_{n=0}^{\infty} \frac{(-\sin^2\theta)^n}{n!}(\zeta + \gamma\sin^2\phi)^n$$
$$= \sum_{n=0}^{\infty} \frac{(-\sin^2\theta)^n}{n!} \sum_{k=0}^{n} \binom{n}{k} \zeta^{n-k}\gamma^k \sin^{2k}\phi \; .$$

Now, it is easily verified that $\int_0^{2\pi} \sin^{2k}\phi \, \mathrm{d}\phi = 2\pi 4^{-k}\binom{2k}{k}$ and that $\int_0^{\pi} \sin^{2n+1}\theta \, \mathrm{d}\theta = \frac{\sqrt{\pi} n!}{(n+1/2)!}$. Hence (S7) becomes (assuming for the moment $\alpha \neq 0$)

$$\bar{S} = e^{-Q_0}\frac{\sqrt{\pi}}{2}\sum_{n=0}^{\infty}\frac{(-\alpha\delta)^n}{(n+1/2)!}\sum_{k=0}^{n}\binom{n}{k}\binom{2k}{k}\left(\frac{\beta-\alpha}{4\alpha}\right)^k . \tag{S8}$$

From the definition of the hypergeometric function ${}_2F_1$ (see II), it is also straightforward to check that the inner sum can be identified as, ${}_2F_1\left(\frac{1}{2}, -n; 1; \frac{\alpha-\beta}{\alpha}\right)$. Thereby we obtain

$$\bar{S} = e^{-Q_0}\frac{\sqrt{\pi}}{2}\sum_{n=0}^{\infty}\frac{(-\alpha\delta)^n}{(n+1/2)!}{}_2F_1\left(\tfrac{1}{2}, -n; 1; \tfrac{\alpha-\beta}{\alpha}\right) \tag{S9}$$

Substituting the value (S1) of Q_0, as well as α, β, and δ, we obtain the first form (8a) when $\alpha \neq 0$, i.e., $a \neq c$. The case $\alpha = a - c = 0$ can be handled explicitly from the start, or through continuity arguments using (S28), in the form $\lim_{x\to 0}(-x)^n {}_2F_1(\frac{1}{2}, -n; 1; \frac{y}{x}) = \frac{(n-1/2)!}{\sqrt{\pi} n!} y^n$, which leads to (8a) when $a = c$ upon recognizing the Taylor series for the error function.

The form (8b) is obtained upon changing the order of summation in Eq. (S8). We obtain

$$\bar{S} = e^{-Q_0}\frac{\sqrt{\pi}}{2}\sum_{k=0}^{\infty}\binom{2k}{k}\left(\frac{\beta-\alpha}{4\alpha}\right)^k\sum_{n=k}^{\infty}\frac{(-\alpha\delta)^n}{(n+1/2)!}\binom{n}{k} ,$$

and from the relation

$$\sum_{n=k}^{\infty}\frac{(-\alpha\delta)^n}{(n+1/2)!}\binom{n}{k} = \frac{(-\alpha\delta)^k}{(k+1/2)!}\,{}_1F_1\left(k+1; k+\tfrac{3}{2}; -\alpha\delta\right)$$

the second form (8b) follows (after renaming the summation index $k \to n$).

Another way the sum (S8) can be rewritten is via the change of summation indices, $m = n - k$, which yields,

$$\bar{S} = e^{-Q_0}\frac{\sqrt{\pi}}{2}\sum_{m=0}^{\infty}\sum_{k=0}^{\infty}\frac{(-\alpha\delta)^m(-1)^k}{(m+k+1/2)!}\binom{m+k}{k}\binom{2k}{k}\left[\frac{\delta(\beta-\alpha)}{4}\right]^k .$$

The identity

$$\sum_{k=0}^{\infty}\frac{\left[\frac{\delta(\alpha-\beta)}{4}\right]^k\binom{2k}{k}\binom{k+m}{k}}{\left(k+m+\frac{1}{2}\right)!} = \frac{{}_2F_2\left(\frac{1}{2}, m+1; 1, m+\frac{3}{2}; \delta(\alpha-\beta)\right)}{(m+\frac{1}{2})!}$$

then gives the form (8c) similarly.

Special cases

All expressions (8) for the powder-averaged signal of course give the same result, and $\bar{S}$ is also invariant under permutations of a, b, c. One can exploit this to derive the special cases where the matrix $\mathbf{D}$ is also axisymmetric, that is, it has two eigenvalues coinciding.

Swapping a and c thanks to the aforementioned invariance, and then putting $b = c$ in Eq. (8a), we get

$$\bar{S} = e^{-ad-2fc}\frac{\sqrt{\pi}}{2}\sum_{n=0}^{a}\frac{[(a-c)(d-f)]^n}{(n+1/2)!},$$

which corresponds to case 3 of *Results* when both $\mathbf{D}$ and $\mathbf{B}$ are axisymmetric. The identity

$$\sum_{n=0}^{\infty}\frac{x^n}{(n+1/2)!} = \frac{e^x \mathrm{erf}\,(\sqrt{x})}{\sqrt{x}} = \frac{e^x \mathrm{erfi}\,(\sqrt{-x})}{\sqrt{-x}}$$

then yields the associated result (7).

The case where $\mathbf{B}$ is not only axisymmetric but also rank-1 follows by putting $f = 0$, which gives Eq. (6).

The simplest case of isotropic $\mathbf{B}$ can be verified as follows: As $\epsilon = 0$ already, isotropy implies $\delta = 0$, and the expressions in (8) readily give Eq. (5), due to all but the $n = 0$ term vanishing in the series expansions.

B. D and B both general

The problem now is to calculate the integral in Eq. (S6), where we no longer assume that $\epsilon = 0$. As a result, the integrand of Eq. (S6) becomes dependent on μ through Q_2, and the μ integration does not drop, contrary to the previous case.

From (S5) and the expression for $R_{\boldsymbol{u}}(\mu)\boldsymbol{v}$, we find that

$$\begin{aligned} Q_2 &= \frac{\epsilon}{2}[\tau + \rho\cos(2\mu) + \sigma\sin(2\mu)], \text{ where} \\ \tau &= \alpha + \beta - \left[\alpha + (\beta-\alpha)\sin^2\phi\right]\sin^2\theta \\ \rho &= -\alpha + \beta + 2(\alpha-\beta)\sin^2\phi + \left[\alpha + (\beta-\alpha)\sin^2\phi\right]\sin^2\theta \\ \sigma &= 2(\alpha-\beta)\sin\phi\cos\phi\cos\theta \end{aligned} \tag{S10}$$

Hence, integrating over μ, we find

$$\begin{aligned} \frac{1}{2\pi}\int_0^{2\pi} e^{-Q_2}\,\mathrm{d}\mu &= \frac{e^{-\frac{\epsilon}{2}\tau}}{2\pi}\int_0^{2\pi} e^{-\frac{\epsilon}{2}[\rho\cos(2\mu)+\sigma\sin(2\mu)]}\,\mathrm{d}\mu \\ &= \frac{e^{-\frac{\epsilon}{2}\tau}}{2\pi}\int_0^{2\pi} e^{-\frac{\epsilon}{2}\sqrt{\rho^2+\sigma^2}\cos(2\mu)}\,\mathrm{d}\mu = e^{-\frac{\epsilon}{2}\tau} I_0\Big(\tfrac{\epsilon}{2}\sqrt{\rho^2+\sigma^2}\Big) \end{aligned} \tag{S11}$$

where we have used $\int_0^{2\pi} e^{\pm A\cos(2\mu)}\,\mathrm{d}\mu = 2\pi I_0(A)$, I_0 being the zeroth order modified Bessel function of the first kind. Inserting (S11) into (S6), the integral is now

$$\bar{S} = \frac{e^{-Q_0}}{4\pi}\int_0^{2\pi}\int_0^{\pi} e^{-\frac{\epsilon}{2}\tau-\delta(\alpha+(\beta-\alpha)\sin^2\phi)\sin^2\theta} I_0\Big(\tfrac{\epsilon}{2}\sqrt{\rho^2+\sigma^2}\Big)\sin\theta\,\mathrm{d}\theta\,\mathrm{d}\phi.$$

For the argument of the exponential function, we have

$$\begin{aligned} &-\tfrac{\epsilon}{2}\tau - \delta(\alpha + (\beta-\alpha)\sin^2\phi)\sin^2\theta \\ &\quad= -\tfrac{\epsilon}{2}(\alpha+\beta) - (\delta + \tfrac{\epsilon}{2})(\alpha + (\beta-\alpha)\sin^2\phi)\sin^2\theta \\ &\quad= -\tfrac{\epsilon}{2}(\alpha+\beta) - (A + B\sin^2\phi)\sin^2\theta \end{aligned} \tag{S12}$$

where $A = (\delta - \frac{\epsilon}{2})\alpha = \frac{1}{2}(a-c)(2d-e-f)$, $B = (\delta - \frac{\epsilon}{2})(\beta - \alpha) = \frac{1}{2}(b-a)(2d-e-f)$. Note that $I_0(\frac{\epsilon}{2}\sqrt{\rho^2+\sigma^2}) = I_0(\frac{|\epsilon|}{2}\sqrt{\rho^2+\sigma^2})$ since I_0 is even.

For the argument of I_0 in (S11) we find that

$$\rho^2+\sigma^2=(\alpha-\beta)^2+2(\beta-\alpha)\left(\alpha-(\alpha+\beta)\sin^2\phi\right)\sin^2\theta+\left(\alpha+(\beta-\alpha)\sin^2\phi\right)^2\sin^4\theta$$

which means that

$$\tfrac{|\epsilon|}{2}\sqrt{\rho^2+\sigma^2}=\sqrt{C+(D+E\sin^2\phi)\sin^2\theta+(F+G\sin^2\phi)^2\sin^4\theta},$$

where $C=\frac{\epsilon^2}{4}(\alpha-\beta)^2=\frac{(e-f)^2}{4}(a-b)^2$, $D=\frac{\epsilon^2}{2}(\beta-\alpha)\alpha=\frac{(e-f)^2}{2}(b-a)(a-c)$, $E=\frac{\epsilon^2}{2}(\alpha^2-\beta^2)=\frac{(e-f)^2}{2}(a-b)(a+b-2c)$, $F=\frac{\epsilon\alpha}{2}=\frac{(e-f)(a-c)}{2}$, and $G=\frac{\epsilon(\beta-\alpha)}{2}=\frac{(e-f)(b-a)}{2}$. Note that $C\geqslant 0$.

Extracting the ϕ- and θ-independent part of (S12), and putting

$$e^{-Q_0'}=e^{-Q_0}e^{-\frac{\epsilon}{2}(\alpha+\beta)}=e^{-\frac{1}{2}(a+b)(e+f)-cd}$$

the integral boils down to

$$\begin{aligned}\bar{S}&=\frac{e^{-Q_0'}}{4\pi}\int_0^{2\pi}\int_0^{\pi}e^{-(A+B\sin^2\phi)\sin^2\theta}I_0(X)\sin\theta\,\mathrm{d}\theta\,\mathrm{d}\phi,\\ X&=\sqrt{C+(D+E\sin^2\phi)\sin^2\theta+(F+G\sin^2\phi)^2\sin^4\theta}\ .\end{aligned}\tag{S13}$$

The idea is now to combine the expansion from I A:

$$e^{-(A+B\sin^2\phi)\sin^2\theta}=\sum_{n=0}^{\infty}\sum_{l=0}^{n}\frac{(-1)^n}{n!}\binom{n}{l}A^{n-l}B^l\sin^{2n}\theta\sin^{2l}\phi\tag{S14}$$

with a Taylor expansion of $I_0(X)$. Namely, with $x=\sin^2\phi, y=\sin^2\theta$ we seek the Taylor expansion

$$I_0(\sqrt{C+(D+Ex)y+(F+Gx)^2y^2})=\sum_{k=0}^{\infty}\sum_{m=0}^{k}q_{mk}x^my^k\tag{S15}$$

where the particular form of the argument implies that the summation over m (for each fixed k) runs from 0 to k. So, for each pair (m,k) in this series and for each (n,l) in the series (S14) we use that

$$\begin{aligned}\frac{1}{4\pi}\int_0^{2\pi}\int_0^{\pi}\sin^{2n}\theta\sin^{2l}\phi\,x^my^k\sin\theta\,\mathrm{d}\theta\,\mathrm{d}\phi&=\frac{1}{4\pi}\int_0^{2\pi}\int_0^{\pi}\sin^{2(n+k)+1}\theta\sin^{2(l+m)}\phi\,\mathrm{d}\theta\,\mathrm{d}\phi\\&=\frac{1}{2}4^{-m-l}\binom{2(l+m)}{l+m}\frac{\sqrt{\pi}(n+k)!}{(n+k+1/2)!}.\end{aligned}$$

This means that (S13) takes the form

$$\bar{S}=\sum_{k=0}^{\infty}\sum_{m=0}^{k}q_{mk}Y_{mk}\ ,\tag{S16}$$

where

$$Y_{mk}=\sum_{n=0}^{\infty}\sum_{l=0}^{n}\frac{(-1)^n}{n!}\binom{n}{l}A^{n-l}B^l\frac{4^{-m-l}}{2}\binom{2(l+m)}{l+m}\frac{\sqrt{\pi}(n+k)!}{(n+k+1/2)!}\ .\tag{S17}$$

This expression is comparable to (S8), which was explicitly summed in various ways to give the different expressions for $\bar{S}$ in Eq. (8). Here, we can proceed in the same way to obtain the three formulas given in Eq. (10), each corresponding to an alternative form $Y_{mk}^{(i)}$.

The first alternative (10c) follows from taking the sum over l, showing that Y_{mk} can be written

$$Y_{mk}^{(1)}(A,B)=\frac{\sqrt{\pi}4^{-m}}{2}\binom{2m}{m}\sum_{n=0}^{\infty}\frac{(-A)^n(k+n)!\,{}_2F_1\left(m+\frac{1}{2},-n;m+1;-\frac{B}{A}\right)}{n!(k+n+1/2)!}\ ,\tag{S18}$$

where we recall $A = \frac{1}{2}(a-c)(2d-e-f)$ and $B = \frac{1}{2}(b-a)(2d-e-f)$. This extends to $A=0$ by continuity via $\lim_{x\to 0} x^n\, {}_2F_1\left(m+\frac{1}{2}, -n; m+1; -\frac{1}{x}\right) = \frac{(m+1/2)_n}{(m+1)_n}$, where $(a)_n$ is the Pochhammer symbol (see II), and leads to the exceptional case in (10c).

Alternatively, rearranging $\sum\limits_{n=0}^{\infty}\sum\limits_{l=0}^{n} = \sum\limits_{l=0}^{\infty}\sum\limits_{n=l}^{\infty}$ and summing over n yields

$$Y_{mk}^{(2)}(A,B) = \frac{\sqrt{\pi}4^{-m}}{2}\sum_{l=0}^{\infty}\frac{(-B)^l(k+l)!\binom{2(l+m)}{l+m}\,{}_1F_1\left(k+l+1; k+l+\frac{3}{2}; -A\right)}{4^l l!\,(k+l+1/2)!}\ , \tag{S19}$$

which is Eq. (10d) after renaming the summation index and substituting the values of A and B.

Finally, one can rearrange the double summation in Eq. (S17) by putting $n=\sigma+l$, yielding the third alternative (10e) after summation over l as

$$Y_{mk}^{(3)}(A,B) = \frac{\sqrt{\pi}4^{-m}}{2}\binom{2m}{m}\sum_{\sigma=0}^{\infty}\frac{(-A)^\sigma(k+\sigma)!\,{}_2F_2\left(m+\frac{1}{2}, k+\sigma+1; m+1, k+\sigma+\frac{3}{2}; -B\right)}{\sigma!\,(k+\sigma+1/2)!}\ , \tag{S20}$$

upon substituting A, B, and renaming the remaining summation index.

The derivation of the the expressions for $\bar{S}$ given in Eq. (10) are thus complete once the form (10b) of the coefficients q_{mk} in Eq. (S16) is demonstrated. These coefficients inherit their form from the relation (S15). We start by recalling that (we will only need $m \in \mathbf{Z}$)

$$I_m(x) = \sum_{n=0}^{\infty}\frac{1}{n!(n+m)!}\left(\frac{x}{2}\right)^{2n+m}. \tag{S21}$$

Hence, I_m is even (odd) when m is even (odd). Again using $\frac{1}{n!}=0$ when n is a negative integer, it also follows that $I_m = I_{-m}$. Moreover, $m=0$ gives $I_0(x) = \sum_{n=0}^{\infty}\frac{1}{n!^2}\left(\frac{x}{2}\right)^{2n}$. Using this and the trinomial expansion, we get

$$\begin{aligned} I_0(\sqrt{C+(D+Ex)y+(F+Gx)^2y^2}) &= \sum_{n=0}^{\infty}\frac{1}{4^n(n!)^2}[C+(D+Ex)y+(F+Gx)^2y^2]^n \\ &= \sum_{n=0}^{\infty}\frac{1}{4^n(n!)^2}\times\sum_{n_1+n_2+n_3=n}\binom{n}{n_1 n_2 n_3}C^{n_1}(D+Ex)^{n_2}y^{n_2}(F+Gx)^{2n_3}y^{2n_3} \end{aligned} \tag{S22}$$

where $\binom{n}{n_1 n_2 n_3} = \frac{n!}{n_1!n_2!n_3!}$. The coefficient (including powers of x) of y^k is given by

$$\begin{cases} n_1+n_2+n_3 = n \\ n_2+2n_3 = k \end{cases},\ \text{i.e.,}\ \begin{cases} n_1 = n-k+n_3 \\ n_2 = k-2n_3 \end{cases}.$$

Replacing n_1, this coefficient is

$$\begin{aligned} &\sum_{n=0}^{\infty}\frac{1}{4^n n!}\sum_{n_3=0}^{k/2}\frac{C^{n-k+n_3}}{(n-k+n_3)!n_2!n_3!}(D+Ex)^{n_2}(F+Gx)^{2n_3} \\ &= \sum_{n_3=0}^{k/2}\left(\frac{\sqrt{C}}{2}\right)^{k-n_3} I_{k-n_3}(\sqrt{C})\frac{C^{-k+n_3}}{n_2!n_3!}(D+Ex)^{n_2}(F+Gx)^{2n_3}. \end{aligned} \tag{S23}$$

where we have used (S21). From this we want to extract the coefficient of x^m. Now,

$$(D+Ex)^{n_2}(F+Gx)^{2n_3} = \sum_{\eta_2=0}^{n_2}\binom{n_2}{\eta_2}D^{n_2-\eta_2}E^{\eta_2}x^{\eta_2}\sum_{\eta_3=0}^{2n_3}\binom{2n_3}{\eta_3}F^{2n_3-\eta_3}G^{\eta_3}x^{\eta_3}, \tag{S24}$$

and hence the coefficient of x^m is obtained when $\eta_2+\eta_3=m$. So, assuming that $\frac{EF}{DG}$ is well defined, and inserting

$\eta_3 = m - \eta_2$, the coefficient of x^m in (S24) is

$$\begin{aligned}
&\sum_{\eta_2=0}^{n_2} \binom{n_2}{\eta_2} D^{n_2-\eta_2} E^{\eta_2} \binom{2n_3}{m-\eta_2} F^{2n_3-m+\eta_2} G^{m-\eta_2} \\
&= D^{n_2} F^{2n_3-m} G^m \sum_{\eta_2=0}^{k-2n_3} \binom{n_2}{\eta_2}\binom{2n_3}{m-\eta_2}\left(\frac{EF}{DG}\right)^{\eta_2} \\
&= D^{k-2n_3} F^{2n_3-m} G^m \sum_{\eta_2=0}^{k-2n_3} \binom{k-2n_3}{\eta_2}\binom{2n_3}{m-\eta_2}\left(\frac{EF}{DG}\right)^{\eta_2},
\end{aligned} \tag{S25}$$

where in the last equation we have used $n_2 = k - 2n_3$.

From the definition of the regularized hypergeometric function (see II) ${}_2\widetilde{F}_1(\cdot)$ it follows that[1]

$$\sum_{\eta_2=0}^{k-2n_3} \binom{k-2n_3}{\eta_2}\binom{2n_3}{m-\eta_2} x^{\eta_2} = x^{m-2n_3}\frac{(k-2n_3)!}{(k-m)!}\,{}_2\tilde{F}_1(m-k,-2n_3;1+m-2n_3;x). \tag{S26}$$

Combining (S23) (with $n_2 = k - n_3$), (S25) and (S26) (with $x = \frac{EF}{DG}$) we find that

$$q_{mk} = \left(\frac{E}{D}\right)^m \left(\frac{D}{2\sqrt{C}}\right)^k \frac{1}{(k-m)!} \sum_{n_3=0}^{k/2} \frac{I_{k-n_3}(\sqrt{C})}{n_3!}\left(\frac{2\sqrt{C}G^2}{E^2}\right)^{n_3} {}_2\tilde{F}_1\left(m-k,-2n_3;1+m-2n_3;\frac{EF}{DG}\right).$$

Using the expressions for C, D, E, F, G, this (with $n_3 = j$) gives precisely the expression (10b) (when $a \neq b$ and $a + b - 2c = 0$) up to the fact that $\sqrt{C}$ contains a modulus: $\sqrt{C} = \frac{1}{2}|e-f| \cdot |a-b|$. However, the parity of I_{k-n_3} together with the powers of $\sqrt{C}$ shows that the modulus may be removed and hence the desired form of q_{mk} is established when $\frac{EF}{DG} \neq 0$, which corresponds to $a \neq b$ and $a + b - 2c \neq 0$.

When $a = b$, this implies that $C = D = E = G = 0$, and inserting this in (S22), we readily get the expression (10b) for the case $a = b$. Moreover, by putting $a = b$ in the formulas (10c–e), they all become (possibly after a summation) $\frac{k!}{(k+1/2)!}{}_1F_1(1+k;\frac{3}{2}+k;\frac{1}{2}(c-a)(2d-e-f))$ so that (8d) follows. The case $a+b-2c = 0$ can be handled by a limit procedure or by using the footnote preceding Eq. (S26). Either way, this establishes (10b) when $a + b - 2c = 0$.

II. SOME HYPERGEOMETRIC FACTS

In this appendix we will give some elementary facts from the extensive field of hypergeometric functions, which are used at various places. Hypergeometric functions are power series $\sum_{n=0}^{\infty} a_n x^n$ where the quotient a_{n+1}/a_n is a rational function of n. We will mostly use the functions (from which the general pattern should be clear)

$${}_1F_1(a;b;x) = \sum_{n=0}^{\infty}\frac{(a)_n}{(b)_n}\frac{x^n}{n!},\ {}_2F_1(a,b;c;x) = \sum_{n=0}^{\infty}\frac{(a)_n(b)_n}{(c)_n}\frac{x^n}{n!},\ {}_2F_2(a,b;c,d;x) = \sum_{n=0}^{\infty}\frac{(a)_n(b)_n}{(c)_n(d)_n}\frac{x^n}{n!}, \tag{S27}$$

where $(a)_n$ is the Pochhammer symbol

$$(a)_n = a(a+1)(a+2)\cdots(a+n-1),$$

which is $\frac{\Gamma(a+n)}{\Gamma(a)}$ if a is not a non-positive integer.

It is immediate that all hypergeometric functions have value 1 when $x = 0$. From the definition (S27), it is easy to see that ${}_2F_1\left(\frac{1}{2},-n;1;x\right)$ are polynomials for $n \in \mathbf{N}$, which are positive and decreasing for negative x. It also follows that ${}_1F_1\left(k+1;k+\frac{3}{2};x\right)$ and ${}_2F_2\left(\frac{1}{2},m+1;1,m+\frac{3}{2};x\right)$ are positive and increasing for $x \geqslant 0$ for all $k, m \in \mathbf{N}$. In addition, there is a relation ${}_1F_1\left(k+1;k+\frac{3}{2};-x\right) = e^{-x}{}_1F_1\left(\frac{1}{2};k+\frac{3}{2};x\right)$ which shows that for any

[1] Valid if $x \neq 0$, otherwise the sum is $\binom{2n_3}{m}$.

$k \in \mathbf{N}$, ${}_1F_1\left(k+1; k+\frac{3}{2}; x\right) > 0$ for all x. It also holds that $\frac{\partial}{\partial x}\,{}_1F_1\left(k+1; k+\frac{3}{2}; x\right) = \frac{(k+1)\,{}_1F_1\left(k+2;k+\frac{5}{2};x\right)}{k+\frac{3}{2}}$, so the conclusion is that for any nonnegative integer k, ${}_1F_1\left(k+1; k+\frac{3}{2}; x\right)$ is positive and also increasing on $\mathbf{R}$. (Similar remarks hold for ${}_2F_2(1/2, m+1; 1, m+3/2; x)$.)

In sections I A and I B, we also use the result (for non-negative integers n, m)

$$\lim_{x\to 0} x^n {}_2F_1(m+\tfrac{1}{2}, -n; m+1; \tfrac{y}{x}) = \frac{(m+1/2)_n}{(m+1)_n}(-y)^n \tag{S28}$$

which is rather immediate from the definition, bearing in mind that ${}_2F_1(m+\frac{1}{2}, -n; m+1; \frac{y}{x})$ is a polynomial of degree n in the variable $\frac{y}{x}$. $m=0$ gives the value $\frac{(1/2)_n}{(1)_n}(-y)^n = \frac{\Gamma(n+1/2)}{\Gamma(1/2)n!}(-y)^n = \frac{(n-1/2)!}{\sqrt{\pi}n!}(-y)^n$. Also, we use the regularized hypergeometric function ${}_2\tilde{F}_1(a,b;c;x)$ which is related to ${}_2F_1(a,b;c;x)$ via

$${}_2\tilde{F}_1(a,b;c;x) = \frac{{}_2F_1(a,b;c;x)}{\Gamma(c)}$$

In the section titled "*Numerical Behaviour*" we saw that for some parameter values (suitably arranged), the series (8d) using the function ${}_1F_1$ gave very efficient summations. In addition, the computation of ${}_1F_1$ for the particular parameter values we need can also be facilitated by the explicit expressions or the recursion formula given below:

$$\begin{aligned}
{}_1F_1\left(1;\tfrac{3}{2};x\right) &= \frac{\sqrt{\pi}e^x \operatorname{erf}\left(\sqrt{x}\right)}{2\sqrt{x}}\ , \\
{}_1F_1\left(2;\tfrac{5}{2};x\right) &= \frac{3\sqrt{\pi}e^x(2x-1)\operatorname{erf}\left(\sqrt{x}\right)}{8x^{3/2}} + \frac{3}{4x}\ , \\
{}_1F_1\left(3;\tfrac{7}{2};x\right) &= \frac{15\left(\sqrt{\pi}e^x\left(4x^2-4x+3\right)\operatorname{erf}\left(\sqrt{x}\right)+2\sqrt{x}(2x-3)\right)}{64x^{5/2}}\ , \\
{}_1F_1\left(4;\tfrac{9}{2};x\right) &= \frac{35(\sqrt{\pi}e^x\left(8x^3-12x^2+18x-15\right)\operatorname{erf}\left(\sqrt{x}\right)}{256x^{7/2}} \\
&+ \frac{+70\sqrt{x}\left(4x^2-8x+15\right))}{256x^{7/2}}\ , \\
{}_1F_1\left(k+2;k+\frac{5}{2};x\right) &= \frac{\left(k+\frac{3}{2}\right)\left(k+\frac{1}{2}\right)}{(k+1)x}\,{}_1F_1\left(k;k+\frac{1}{2};x\right) \\
&- \frac{\left(k+\frac{3}{2}\right)\left(k+\frac{1}{2}-x\right)}{(k+1)x}\,{}_1F_1\left(k+1;k+\frac{3}{2};x\right)\ .
\end{aligned}$$

III. SERIES EXPANSION OF $\langle e^{-\lambda\,\mathrm{tr}(\mathbf{R}^\intercal\mathbf{D}\mathbf{R}\widetilde{\mathbf{B}})}\rangle_{\mathbf{R}}$

In this appendix we comment on the derivation of the coefficients c_1, c_2, c_3 in the expansion given in Eq. (16). So, with $\bar{S}_{\mathbf{D},\widetilde{\mathbf{B}}}(\lambda) = \langle e^{-\lambda\,\mathrm{tr}(\mathbf{R}^\intercal\mathbf{D}\mathbf{R}\widetilde{\mathbf{B}})}\rangle_{\mathbf{R}}$, we seek $\bar{S}_{\mathbf{D},\widetilde{\mathbf{B}}}(\lambda) = 1 + c_1(\mathbf{D},\widetilde{\mathbf{B}})\lambda + c_2(\mathbf{D},\widetilde{\mathbf{B}})\lambda^2 + c_3(\mathbf{D},\widetilde{\mathbf{B}})\lambda^3 + \mathcal{O}(\lambda^4), \lambda \to 0$. We assume that $\mathbf{D}$ has eigenvalues a, b, c and that $\widetilde{\mathbf{B}}$ has eigenvalues d, e, f. From the definition of $\bar{S}$, it is clear that c_k is a homogeneous polynomial in a, b, c, d, e, f of degree $2k$ and that for fixed d, e, f, c_k is a homogeneous symmetric polynomial in a, b, c of degree k (and vice versa with the roles of a, b, c and d, e, f changed). For instance, c_1 must be proportional to $(a+b+c)(d+e+f) = \mathrm{tr}(\mathbf{D})\,\mathrm{tr}(\widetilde{\mathbf{B}})$. To derive the coefficients c_i, one can either use Eq. (10) with the replacement $d \to \lambda d, e \to \lambda e, f \to \lambda f$, and then use the known expansions w.r.t. λ of all involved functions. An alternative is to start with Eq. (S6). Reusing the notation and inserting $e^{-Q_0} = e^{cf-c(d+e)-f(a+b)}$, we therefore look at $e^{cf-c(d+e)-f(a+b)-Q_1-Q_2}$ where we express Q_1 given by (S4) and Q_2 given by (S10) in terms of a, b, c, d, e, f. By the same replacement $d \to \lambda d, e \to \lambda e, f \to \lambda f$, it is then straightforward to to look at the series expansion w.r.t λ of the exponential function first, and then perform the integrals as indicated in Eq. (S6). Either way it is straightforward,

but a bit tedious, to get

$$
\begin{aligned}
3c_1 &= -(a+b+c)(d+e+f) \\
30c_2 &= \left(a^2+b^2+c^2\right)\left(3d^2+2d(e+f)+3e^2+2ef+3f^2\right) \\
&\quad + 2(a(b+c)+bc)\left(d^2+4d(e+f)+e^2+4ef+f^2\right) \\
210c_3 &= -2\left(a^3+b^3+c^3\right)\left(d^3-5d^2(e+f)-d\left(5e^2+8ef+5f^2\right)+e^3-5e^2f-5ef^2+f^3\right) \\
&\quad -(a+b+c)\left(a^2+b^2+c^2\right)\left(3d^3+13(e+f)(d^2+e+f)+d\left(13(e^2+f^2)+18ef\right)+3(e^3+f^3)\right) \\
&\quad -2abc\left(d^3+9d^2(e+f)+d\left(9e^2+48ef+9f^2\right)+e^3+9e^2f+9ef^2+f^3\right)
\end{aligned}
$$

and then compare with Eq. (16) to see that the expressions coincide.

IV. SOLUTIONS TO EQUATION (20)

In this appendix, we address in more detail our claim that there are situations where using a general measurement tensor $\mathbf{B}$ is crucial in determining the diffusive properties of the specimen. Namely, suppose we are given a specimen with two substances with diffusivity matrices $\mathbf{D} \sim \left(\begin{smallmatrix} a & 0 & 0 \\ 0 & b & 0 \\ 0 & 0 & c \end{smallmatrix}\right)$ and $\widetilde{\mathbf{D}} \sim \left(\begin{smallmatrix} q & 0 & 0 \\ 0 & 0 & 0 \\ 0 & 0 & 0 \end{smallmatrix}\right)$ which occur in unknown proportions p and $1-p$. We define $x = \mathrm{tr}(\mathbf{D})$, $y = \mathrm{tr}(\mathbf{D}^2)$, $z = \mathrm{tr}(\mathbf{D}^3)$. By the procedure described in the section titled "*Estimation of* $\mathbf{D}$ *from the power series expansion of* $\bar{S}$" and using the measurement tensor $\widetilde{\mathbf{B}} = \left(\begin{smallmatrix} \delta & 0 & 0 \\ 0 & \epsilon & 0 \\ 0 & 0 & 0 \end{smallmatrix}\right)$, we know the values of

$$(\delta+\epsilon)(px+(1-p)q)\ , \tag{S29a}$$

$$(\delta^2+\epsilon^2)(p\left(x^2+2y\right)+(1-p)3q^2)+\delta\epsilon(p(4x^2-2y)+(1-p)2q^2)\ , \tag{S29b}$$

$$(\delta^3+\epsilon^3)\left(p(x^3+6xy+8z)+(1-p)15q^3\right)+(\delta^2\epsilon+\delta\epsilon^2)(p(9x^3+12xy-12z)+(1-p)9q^3)\ , \tag{S29c}$$

for different values of δ and ϵ. Using an isotropic measurement tensor, we also know the values of

$$px+(1-p)q\ , \tag{S30a}$$

$$px^2+(1-p)q^2\ , \tag{S30b}$$

$$px^3+(1-p)q^3\ . \tag{S30c}$$

By varying δ and ϵ, we see that (S29b) and (S29c) contain two independent expressions each, and hence (S29a-c) will give five expressions. We also see that the quantity (S30a) is a special case of (S29a). Furthermore, since the expression in (S29b) for $\delta=0$ subtracted from the same expression for $\delta=\epsilon$ is $=5\epsilon^2(px^2+(1-p)q^2)$, the quantity in (S30b) is also redundant. In total, we get six equations:

$$px+(1-p)q=k_1\ , \tag{S31a}$$

$$p\left(x^2+2y\right)+(1-p)3q^2=k_2\ , \tag{S31b}$$

$$p\left(x^2-y\right)=k_3\ , \tag{S31c}$$

$$p(x^3+6xy+8z)+(1-p)15q^3=k_4\ , \tag{S31d}$$

$$p(x^3+xy-2z)=k_5\ , \tag{S31e}$$

$$px^3+(1-p)q^3=m_1, \tag{S31f}$$

which follow from (S29) and (S30c) as follows. Writing (LS31a) for the left hand side of equation (S31a) et cetera, we readily find that (LS31a)=(S29a)/$(\delta+\epsilon)$, (LS31b)=$\frac{1}{\epsilon^2}$(S29b)$|_{\delta=0}$, (LS31c)=$\frac{3}{10\epsilon^2}$(S29b)$|_{\delta=\epsilon}$ - $\frac{4}{5\epsilon^2}$(S29b)$|_{\delta=0}$, (LS31d)=$\frac{1}{\epsilon^3}$(S29c)$|_{\delta=0}$, (LS31e)=$\frac{5}{84\epsilon^3}$(S29c)$|_{\delta=\epsilon}$ - $\frac{4}{21\epsilon^3}$(S29c)$|_{\delta=0}$ and (LS31f)=(S30c). Thus, the first five equations above come from the measurement tensor $\widetilde{\mathbf{B}} = \left(\begin{smallmatrix} \delta & 0 & 0 \\ 0 & \epsilon & 0 \\ 0 & 0 & 0 \end{smallmatrix}\right)$ while (S31f) comes from isotropic measurements. To recollect, by varying ϵ and δ in the measurement tensor $\widetilde{\mathbf{B}} = \left(\begin{smallmatrix} \delta & 0 & 0 \\ 0 & \epsilon & 0 \\ 0 & 0 & 0 \end{smallmatrix}\right)$, and by using isotropic measurements, we can determine the left hand sides in equation (S31), which are quantities denoted $k_1, \ldots k_5, m_1$. From the setup of the problem, we know that $0 \leqslant p \leqslant 1$ and $q, a, b, c \geqslant 0$.

Given the values $k_1, \ldots k_5, m_1$, to what extent do these determine p, q, a, b, c? It generically holds: The set of equations (S31a)–(S31e) has two sets of solutions $\{p, q, x, y, z\}$ and $\{P, Q, X, Y, Z\}$ while the inclusion of (S31f) makes

the solution set unique. As an imprecise statement, it also holds that in "most" cases, the second solution set $\{P, Q, X, Y, Z\}$ is ruled out out as unphysical. This can be due to that P is outside the interval $[0, 1]$, some of the quantities Q, X, Y, Z are negative, or that the eigenvalues belonging to a matrix $\hat{\mathbf{D}}$ with $X = \mathrm{tr}(\hat{\mathbf{D}})$, $Y = \mathrm{tr}(\hat{\mathbf{D}}^2)$, $Z = \mathrm{tr}(\hat{\mathbf{D}}^3)$ are complex. However, there are values of p, q, a, b, c where the second solution $\{P, Q, X, Y, Z\}$ corresponds to a realistic specimen mixture.

We find the generic solutions to Eq. (S31a)–(S31e) as follows. (S31c) gives $y = x^2 - \frac{k_3}{p}$ and inserted in (S31e) we get $z = x^3 - \frac{k_3 x + k_5}{2p}$. Next, (S31a) gives $q = \frac{px - k_1}{p-1}$ and inserting these values in (S31b), we find that $p = \frac{3k_1^2 - k_2 - 2k_3}{6k_1 x - k_2 - 2k_3 - 3x^2}$. Finally, with these values inserted in (S31d), we get an equation for x, namely

$$15\left(3k_1^2 - k_2\right)x^2 - 3(5k_1(k_2 + 4k_3) - (k_4 + 4k_5))x - 3k_1(k_4 + 4k_5) + 5(k_2 + 2k_3)^2 = 0.$$

Generically, this equation has two solutions x, X, which are both real (since one is known to be real), and hence two solutions sets are produced. (There are a number of special cases that need attention; e.g., $p = 0, 1$, $3k_1^2 - k_2 - 2k_3 = 0$, $3k_1^2 - k_2 = 0$. For instance, p is obviously non-unique when $\mathbf{D} = \widetilde{\mathbf{D}}$. Each such case is straightforward, but also questionable from a measurement point of view.) As mentioned above, most often the "second" solution $\{P, Q, X, Y, Z\}$ can be ruled out as unphysical. On the other hand, starting with $p = 9/10, q = 5, a = 1/10, b = 1, c = 3$ (so that $x = 41/10$, $y = 1001/100$, $z = 28001/1000$) then also $\{P, Q, X, Y, Z\} = \{178409449/178940890,\ 2467/270,\ 55769/13357,\ 10075315571/892047245,\ 4243907217264727/119150750514650\}$ is a "physical" solution corresponding to a matrix with eigenvalues $\approx \{.181,\ .715,\ 3.279\}$. However, invoking the measurement with the isotropic matrix, i.e., Eq. (S31f), x is determined uniquely since one easily finds, using (S31c)–(S31f) that $x = \frac{15m_1 - k_4 - 4k_5}{10k_3}$.

V. PROOF OF THEOREM 1

We use Eq. (8b) with $c = e = f = 0$. Then $\bar{S} = \bar{S}_{a,b,0}(d) = \sum\limits_{n=0}^{\infty} \frac{[(a-b)d]^n}{(2n+1)n!}\, {}_1F_1\big(n+1; n+\frac{3}{2}; -ad\big)$. The case $a = b$ is already accounted for, and without loss of generality, we can assume that $a > b$. From II we use ${}_1F_1\left(n+1; n+\frac{3}{2}; -x\right) = e^{-x}\, {}_1F_1\left(\frac{1}{2}; n+\frac{3}{2}; x\right)$. Writing $(a-b)d = (1 - \frac{b}{a})ad = (1-\alpha)x$ with $x = ad$ and $0 < \alpha = b/a < 1$, the statement of Theorem 1 reads

$$\lim_{x\to\infty} \frac{1}{a} \sum_{n=0}^{\infty} \frac{(1-\alpha)^n x^{n+1}}{(2n+1)n!} e^{-x}\, {}_1F_1\left(\frac{1}{2}; n+\frac{3}{2}; x\right) = \frac{1}{2\sqrt{ab}}. \tag{S32}$$

From Ref. 3, we find that ${}_1F_1\left(\frac{1}{2}; n+\frac{3}{2}; x\right) = \frac{(n+\frac{1}{2})!}{\sqrt{\pi}} e^x x^{-n-1}(1 + \mathcal{O}(\frac{1}{x}))$ as $x \to \infty$. If we can justify changing the limit and the summation in Eq. (S32), we get

$$\frac{1}{a}\sum_{n=0}^{\infty} \lim_{x\to\infty} \frac{(1-\alpha)^n x^{n+1}}{(2n+1)n!} e^{-x} \frac{(n+\frac{1}{2})!}{\sqrt{\pi}} e^x x^{-n-1}(1 + \mathcal{O}(\tfrac{1}{x})) = \frac{1}{a}\sum_{n=0}^{\infty} \frac{(1-\alpha)^n}{(2n+1)n!} \frac{(n+\frac{1}{2})!}{\sqrt{\pi}} = \frac{1}{a}\frac{1}{2\sqrt{\alpha}} = \frac{1}{2\sqrt{ab}}.$$

Here we have used that (for $|t| < 1$) $\frac{1}{\sqrt{1-t}} = \sum\limits_{n=0}^{\infty} (-1)^n \binom{-1/2}{n} t^n$ with $t = 1 - \alpha$ and observed[2] that $\frac{(-1)^n}{2}\binom{-1/2}{n}$ indeed equals $\frac{(n+\frac{1}{2})!}{\sqrt{\pi}(2n+1)n!}$ for $n = 0, 1, 2, \ldots$. Hence Theorem 1 is proven if we can change summation and limit in Eq. (S32). To see that this is allowed, we define the functions $f_n(x) = \frac{x^{n+1}}{(2n+1)n!} e^{-x}\, {}_1F_1\left(\frac{1}{2}; n+\frac{3}{2}; x\right)$ and consider[3] $\sum\limits_{n=0}^{\infty} (1-\alpha)^n f_n(x)$. It is enough to prove that this series converges uniformly for $x \geqslant 0$, and this will be the case if $\sum\limits_{n=0}^{\infty} (1-\alpha)^n M_n$ converges, where $M_n = ||f_n||_\infty = \sup_{x\geqslant 0} |f_n(x)|$.

To study M_n we use[3] the integral representation ${}_1F_1\left(\frac{1}{2}; n+\frac{3}{2}; x\right) = \frac{\Gamma(n+\frac{3}{2})}{\sqrt{\pi} n!} \int_0^1 e^{xt} \frac{(1-t)^n}{\sqrt{t}} \mathrm{d}t$, so that (after a slight simplification)

$$f_n(x) = \frac{(n-\frac{1}{2})!}{2\sqrt{\pi} n!} \frac{x^{n+1} e^{-x}}{n!} \int_0^1 \frac{e^{xt}}{\sqrt{t}} (1-t)^n \mathrm{d}t\ ,$$

---

[2] For this observation, the reflection formula $\Gamma(z)\Gamma(1-z) = \pi/\sin(\pi z)$ for non integer z is helpful.
[3] The factor $1/a$ is irrelevant.

where we also put $c_n = \frac{(n-\frac{1}{2})!}{2\sqrt{\pi}n!}$ and $g_n(x) = \frac{x^{n+1}e^{-x}}{n!}\int\limits_0^1 \frac{e^{xt}}{\sqrt{t}}(1-t)^n\mathrm{d}t$. Next, we write

$$g_n(x) = \frac{x^{n+1}e^{-x}}{n!}\int\limits_0^1 \frac{e^{xt}}{\sqrt{t}}(1-t)^n\mathrm{d}t = g_{1n}(x) + g_{2n}(x) = \frac{x^{n+1}e^{-x}}{n!}\int\limits_0^1 \frac{1}{\sqrt{t}}(1-t)^n\mathrm{d}t + \frac{x^{n+1}e^{-x}}{n!}\int\limits_0^1 \frac{e^{xt}-1}{\sqrt{t}}(1-t)^n\mathrm{d}t.$$

Since $\int\limits_0^1 \frac{1}{\sqrt{t}}(1-t)^n\mathrm{d}t = \frac{\sqrt{\pi}n!}{(n+1/2)!}$, we find that $g_{1n}(x) = \frac{\sqrt{\pi}x^{n+1}e^{-x}}{(n+1/2)!}$, which attains its maximum (for $x \geqslant 0$) at $x = n+1$ and hence $||g_{1n}||_\infty = \frac{\sqrt{\pi}(n+1)^{n+1}e^{-(n+1)}}{(n+1/2)!}$. To estimate g_{2n}, we need an inequality. First, with $\phi(v) = (1+v)(1-e^{-v})-2v$ it is easy to see that $\phi(v) \leqslant 0$ for $v \geqslant 0$. This means that (for $v \geqslant 0$) $1-e^{-v} \leqslant 2\frac{v}{1+v} \leqslant 2\sqrt{\frac{v}{1+v}}$ and finally $\frac{e^v-1}{\sqrt{v}} \leqslant \frac{2e^v}{\sqrt{1+v}}$. g_{2n} can now be estimated through

$$g_{2n}(x) = \frac{x^{n+1}e^{-x}}{n!}\int\limits_0^1 \sqrt{x}\frac{e^{xt}-1}{\sqrt{xt}}(1-t)^n\mathrm{d}t \leqslant \frac{x^{n+1}e^{-x}}{n!}\int\limits_0^1 \sqrt{x}\frac{2e^{xt}}{\sqrt{1+xt}}(1-t)^n\mathrm{d}t.$$

Splitting the integral into two, we first get

$$\begin{aligned}
\frac{x^{n+1}e^{-x}}{n!}\int\limits_{1/2}^1 \frac{2\sqrt{x}}{\sqrt{1+xt}}e^{xt}(1-t)^n\mathrm{d}t &\leqslant \frac{x^{n+1}e^{-x}}{n!}\int\limits_{1/2}^1 2\sqrt{2}e^{xt}(1-t)^n\mathrm{d}t \\
&= \frac{2\sqrt{2}x^{n+1}}{n!}\int\limits_{1/2}^1 e^{x(t-1)}(1-t)^n dt \\
&= \frac{2\sqrt{2}x^{n+1}}{n!}\int\limits_0^{1/2} e^{-xs}s^n\mathrm{d}s \\
&\leqslant \frac{2\sqrt{2}x^{n+1}}{n!}\int\limits_0^{\infty} e^{-xs}s^n\mathrm{d}s \\
&= \frac{2\sqrt{2}x^{n+1}}{n!}\frac{n!}{x^{n+1}} = 2\sqrt{2}.
\end{aligned}$$

For the remaining part, we get

$$\begin{aligned}
\frac{x^{n+1}e^{-x}}{n!}\int\limits_0^{1/2} \frac{2\sqrt{x}}{\sqrt{1+xt}}e^{xt}(1-t)^n\mathrm{d}t &\leqslant \frac{2x^{n+1}e^{-x}}{n!}\int\limits_0^{1/2} \sqrt{x}e^{xt}(1-t)^n\mathrm{d}t \\
&= \frac{2x^{n+3/2}}{n!}\int\limits_0^{1/2} e^{x(t-1)}(1-t)^n\mathrm{d}t \\
&= \frac{2\sqrt{2}}{n!}\int\limits_{x/2}^{x} \sqrt{\frac{x}{2}}e^{-v}v^n\mathrm{d}t \\
&\leqslant \frac{2\sqrt{2}}{n!}\int\limits_{x/2}^{x} e^{-v}v^{n+1/2}\mathrm{d}t \\
&\leqslant \frac{2\sqrt{2}}{n!}\int\limits_0^{\infty} e^{-v}v^{n+1/2}\mathrm{d}t \\
&= \frac{2\sqrt{2}(n+1/2)!}{n!}\ .
\end{aligned}$$

Collecting terms, we see that

$$M_n \leqslant \frac{(n-\frac{1}{2})!}{2\sqrt{\pi}n!}\left(\frac{\sqrt{\pi}(n+1)^{n+1}e^{-(n+1)}}{(n+1/2)!}+2\sqrt{2}+\frac{2\sqrt{2}(n+1/2)!}{n!}\right).$$

By a straightforward use of Stirling's formula in the form[4] $z! = \Gamma(z+1) = \sqrt{2\pi z}\left(\frac{z}{e}\right)^z (1+\mathcal{O}(\frac{1}{z}))$ as $z\to\infty$, we see that $\frac{(n-\frac{1}{2})!}{2\sqrt{\pi}n!} = \mathcal{O}(\frac{1}{\sqrt{n}})$, $\frac{\sqrt{\pi}(n+1)^{n+1}e^{-(n+1)}}{(n+1/2)!} = \mathcal{O}(1)$ and $\frac{2\sqrt{2}(n+1/2)!}{n!} = \mathcal{O}(\sqrt{n})$, all as $n\to\infty$. In total, $M_n = \mathcal{O}(1)$ as $n\to\infty$ so that $\sum_{n=0}^{\infty}(1-\alpha)^n M_n$ converges (since $0<\alpha<1$). This concludes the proof.

VI. NUMERICAL ESTIMATION OF THE AVERAGE SIGNAL

Alternatively to the formulas presented in the main text, one may consider performing the average over rotations in Eq. (2) numerically. However, such approaches do not tolerate the eigenvalues of the matrix $\mathbf{D}$ (or $\mathbf{B}$) being too disparate very well, as we discuss here after sketching how such numerical averages might work.

The most direct way to obtain the average (2) numerically is to rewrite it in the form of a sample average,

$$\bar{S} \approx \frac{1}{N}\sum_{n=1}^{N} e^{-\mathrm{tr}(\mathbf{D}\mathbf{R}_n^{\intercal}\mathbf{B}\mathbf{R}_n)},\ \{\mathbf{R}_n\}_n \subset \mathrm{SO}(3)\ , \tag{S33}$$

over the set of all rotations. Since SO(3) is compact, there is no problem in "covering" SO(3), but with all weights being $1/N$, we ideally would need a uniform sampling. This is a nontrivial task and an appropriate sampling approach, which is inspired by the integration steps leading to the angular average (S6), is the following. For a given number M, one takes M quasi-uniformly distributed points/vectors $\hat{\boldsymbol{u}}_m$ on S^2. Any two neighboring points determine a typical angular distance $\Delta\alpha$. For any $\hat{\boldsymbol{u}}_m$, one considers a family of rotations around $\hat{\boldsymbol{u}}_m$: $\{\mathbf{R}_{m,\hat{\boldsymbol{u}}_m}(\mu_k)\}_{k=1}^K$ where K is chosen so that the rotation angles $\{\mu_k\}_{k=1}^K$ are evenly distributed on the unit circle and have separation $\sim\Delta\alpha$. In total this gives $N = MK$ rotation matrices through the combinations $\mathbf{R}_{mk} = \mathbf{R}_{m,\hat{\boldsymbol{u}}_m}(\mu_k)\mathbf{R}_m$.

M	4	8	16	32	64	128	256	512	1024
N	12	32	96	288	832	2304	6400	18432	51200

TABLE S1. The number of elements, N, in SO(3) as a function of M, the number of elements chosen in S^2.

As an example, we computed the average (S33) using the parameter values from Figures 1 and 2 in the main text for the sample-counts (N) in table S1. These values, S_N, are compared with $\bar{S}$ computed via the analytical formulas (10), and in figure S1 the relative error is displayed. For the parameters chosen, the relative error is of order 1% when the number of sample points in SO(3) is 6400.

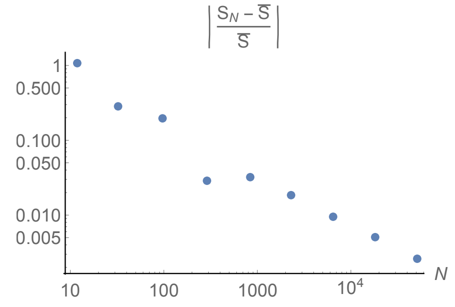

FIG. S1. Relative error when comparing S_N, the powder average computed via sampling in SO(3), with $\bar{S}$, the powder average calculated through the series in Eq. (10), as a function of the sample-count N. A relative error of $\sim 1\%$ is achieved with 6400 sample points in SO(3). The two sets of eigenvalues used, $\{0.1, 0.2, 3\}$ and $\{6, 0.3, 0.7\}$.

[4] Here, z need not be an integer.

The sampling scheme above is in a sense equivalent to a straightforward numerical evaluation of the integral in Eq. (S6) as a discretized Riemann integral. Instead of resorting to numerical evaluation at that stage, an improved alternative would be to perform it after the reduction of the three-fold integral of Eq. (S6) to the two-fold integral of Eq. (S13). With the eigenvalues for $\mathbf{D}$ and $\mathbf{B}$ employed in Figure S1, the integrand from (S13) is shown in Figure S2a. With these parameter values, the computation is straightforward, and easily gives $\bar{S}$. However, other parameter values may result in a badly-behaved integrand, making this approach less useful. Surely enough, this caveat applies to the sampling approach as well, as it can be viewed as the numerical evaluation of the three-fold integral in Eq. (S6) that leads to (S13). We discuss a particular choice of $\mathbf{D}$ and $\mathbf{B}$ below as a demonstration.

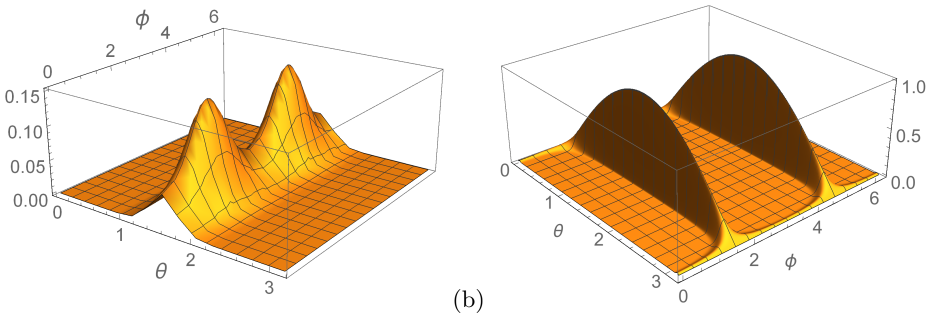

(a) (b)

FIG. S2. $\bar{S}$ is the integral of the functions shown above. (a) With the parameter values as before. This particular choice of parameters leads to a straightforward numerical integration. (b) Both $\mathbf{D}$ and $\mathbf{B}$ have eigenvalues $\{30, 0, 0\}$. Because of the almost-discontinuous behavior of the integrand, the numerical integration is non-trivial.

Consider the case when $\mathbf{D}$ and $\mathbf{B}$ have the same set of eigenvalues: $\{\lambda, 0, 0\}$ (with $\lambda > 0$). Then Eq. (6) applies and $\bar{S} = \frac{\sqrt{\pi}}{2}\frac{\mathrm{erf}(\lambda)}{\lambda}$. When $\lambda = 30$, this gives $\bar{S} \approx 0.0295409$. However, plotting the integrand in (S13), using $a = d = 30, b = c = e = f = 0$, we get the graph in Figure S2b where most of the "mass" producing the volume $\bar{S} \approx 0.0295409$ is distributed along two thin "walls". The integrand is continuous, but has features close to discontinuities, and for that reason, the numerical integration is non-trivial. In the context of the sample average in SO(3), the same problem appears as only a few rotation matrices $\mathbf{R}_n$ making $e^{-\mathrm{tr}(\mathbf{D}\mathbf{R}_n^\intercal \mathbf{B}\mathbf{R}_n)}$ contribute substantially to the sum (S33).

[1]H. Hopf, Mathematische Annalen **104**, 637 (1931).

[2]D. W. Lyons, Mathematics Magazine **76**, 87 (2003).

[3]M. Abramowitz and I. A. Stegun, *Handbook of Mathematical Functions: With Formulas, Graphs, and Mathematical Tables* (Dover Publications, New York, 1977).
